# Postprandial Responses to Meals Enriched With Canola or Coconut Oil in Men and Women With a Risk Phenotype for Cardiometabolic Diseases: A Randomized Crossover Trial

**DOI:** 10.1002/mnfr.70147

**Published:** 2025-06-19

**Authors:** Hannah F. Kienēs, Christina Diekmann, Tim Schiemann, Carolin Wiechmann, Christina Kopp, Birgit Stoffel‐Wagner, Martin Coenen, Robert Németh, Sarah Egert

**Affiliations:** ^1^ Institute of Nutritional and Food Science Nutritional Physiology University of Bonn Bonn Germany; ^2^ Institute of Clinical Chemistry and Clinical Pharmacology University Hospital Bonn Bonn Germany; ^3^ Institute of Medical Biometry Informatics and Epidemiology University Hospital Bonn Bonn Germany

**Keywords:** arterial stiffness, canola oil, cardiometabolic risk, coconut oil, triglyceride response

## Abstract

We investigated the metabolic response to meals with canola or coconut oil (rich in unsaturated vs. rich in saturated fatty acids [FAs]). Although the longer‐term metabolic effects of these fats are well evidenced, their postprandial effects remain inconclusive. In this randomized crossover trial, 29 participants with increased cardiometabolic risk consumed four isoenergetic meals containing 25 or 50 g (low‐fat meals [LFMs], high‐fat meals [HFMs]) of canola or coconut oil. Blood samples for analysis of triglycerides (TGs), glucose, insulin, nonesterified FAs (NEFAs), IL‐6, and individual FAs were collected in the fasting state and 6 h postprandially (every 0.5–1 h). The incremental areas under the curves (iAUCs) of TGs and IL‐6 were higher after canola than after coconut oil. Concentrations of lauric and myristic acid were higher after coconut oil, while concentrations of oleic, linoleic, and α‐linolenic acid were higher after canola oil. The TG iAUC was higher after HFMs than after corresponding LFMs. NEFAs decreased more after LFMs than after HFMs. The glucose and insulin iAUCs were higher after LFMs than after HFMs. Canola and coconut oil induced different metabolic responses. The manner and strength of the postprandial effects differed depending on the parameter.

## Graphical abstract

In this randomized crossover study, 29 participants with increased cardiometabolic risk consumed four isoenergetic, isonitrogenous meals containing 25 or 50 g of canola or coconut oil. Blood samples (e.g., for analysis of lipid, glucose, and insulin metabolism) were collected in the fasting state and 0.5, 1.0, 1.5, 2.0, 3.0, 4.0, 5.0, and 6.0 h postprandially. Besides, vascular function was assessed by measuring arterial stiffness (pulse wave velocity, augmentation index) before the test meals and 2.0, 4.0, and 6.0 h postprandially.

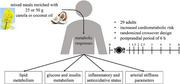

**Abbreviations**
AIx: augmentation index
ANOVA: analysis of variance
BP: blood pressure
CRP: C‐reactive protein
CVD: cardiovascular disease
FA: fatty acid
HFM: high‐fat meal
HOMA‐IR: homeostasis model assessment for insulin resistance
iAUC: incremental area under the curve
LFM: low‐fat meal
MUFA: monounsaturated fatty acid
NEFA: nonesterified fatty acid
PUFA: polyunsaturated fatty acid
PWV_c‐f_
: carotid‐femoral pulse wave velocity
SFA: saturated fatty acid
TEAC: trolox equivalent antioxidative capacity
TG: triglyceride

## 1 Introduction

Current dietary guidelines and specialized societies (e.g., the American Heart Association) recommend that dietary saturated fatty acids (SFAs) are replaced by unsaturated fatty acids (FAs) to lower the incidence of cardiovascular diseases (CVDs) [1, 2, 3]. A versatile fat source rich in unsaturated FAs is canola oil, which has a high content of monounsaturated fatty acids (MUFAs; ∼63 g/100 g) and polyunsaturated fatty acids (PUFAs; ∼25 g/100 g, ∼7.5 g/100 g α‐linolenic acid) [4]. Mainly due to its FA composition, canola oil effectively lowers CVD‐related risk factors; for example, a regular consumption of canola oil reduces fasting concentrations of total and LDL cholesterol [5, 6, 7, 8, 9]. Furthermore, canola oil‐based diets have been shown to improve glucose tolerance and insulin sensitivity [5]. In contrast, regular consumption of coconut oil increases fasting concentrations of total and LDL cholesterol [1, 10, 11], an effect attributed to its high content of SFAs (∼83 g/100 g [12]), especially of lauric, myristic, and palmitic acid (per 100 g coconut oil: ∼42, 17, and 9 g [12]) [13, 14, 15, 16]. These three FAs inhibit LDL receptor activity, enhance LDL cholesterol production, and increase the concentration of LDL cholesterol in mechanistic studies [17].

Variations in the dietary FA composition influence blood parameters associated with cardiovascular risk immediately after food intake [18]. Among acute risk markers, the postprandial triglyceride (TG) concentration plays a central role because nonfasting TGs are an independent risk factor for coronary artery disease and cardiovascular events [19, 20, 21]. Two meta‐analyses showed that the postprandial TG response to meals enriched with unsaturated FAs is lower than that to SFA‐rich meals, at least if the postprandial phase lasts 8 h [18, 22]. In this context, studies comparing the acute effects of coconut oil with MUFA‐ and/or PUFA‐rich meals on the TG concentration provide inconsistent results [23, 24, 25, 26, 27, 28, 29, 30]. The aim of this study was to compare the acute effects of canola and coconut oil in adults with increased cardiometabolic risk (e.g., abdominal obesity and advanced age), because these individuals have a more pronounced postprandial metabolic response [31, 32, 33, 34, 35, 36]. In addition to our primary outcome TG concentration, we analyzed several further metabolic parameters and vascular function by measuring arterial stiffness to comprehensively understand the postprandial effects of canola and coconut oil. Both plant oils were administered in mixed meal challenges to increase the practical applicability.

## 2 Experimental Section

### 2.1 Participants

**FIGURE 1:**
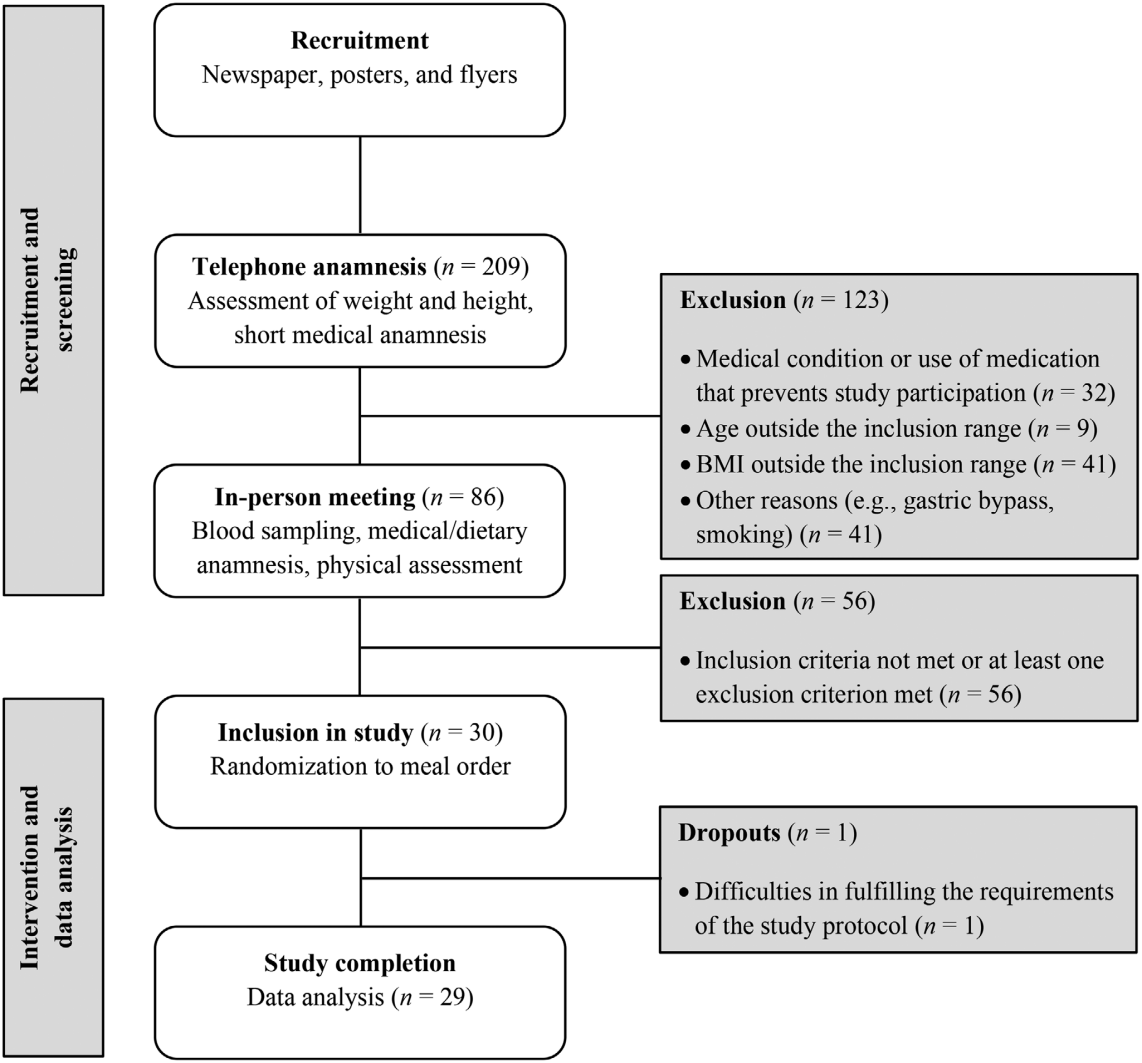
Flowchart of inclusion and exclusion of participants.

This study was conducted at the Institute of Nutritional and Food Science, Nutritional Physiology at the University of Bonn. Volunteers were recruited from Bonn (Germany) and the surrounding area via advertisement in the local newspaper, posters, and flyers. Out of 209 individuals who participated in telephone anamnesis, 86 volunteers attended a screening (Figure 1). This 1‐h screening session comprised (1) fasting blood sampling, analyzed for serum creatinine, total bilirubin, gamma‐glutamyl transferase, alanine transaminase, aspartate transaminase, lipase, serum lipids and lipoproteins, plasma glucose, serum C‐reactive protein (CRP), and blood count; (2) survey of the participant's medical history and dietary habits; and (3) physical assessments, including measurement of height and weight, waist and hip circumference, fat and fat‐free mass, resting blood pressure (BP), and heart rate.

The following inclusion criteria were applied to identify individuals at increased cardiometabolic risk: 60–80 years; overweight or obesity (BMI 27–34.9 kg/m^2^); visceral adiposity (waist circumference ≥ 94 cm for men and ≥ 80 cm for women); two or more of the four characteristics of metabolic syndrome, namely, dyslipidemia (serum TGs ≥ 1.7 mmol/L and/or serum HDL cholesterol < 1.03 mmol/L for men and < 1.29 mmol/L for women), increased resting BP (systolic BP ≥ 130 mmHg and/or diastolic BP ≥ 85 mmHg), and increased plasma glucose (≥ 5.6 mmol/L) [37]. Metabolic syndrome refers to the simultaneous occurrence of several cardiometabolic risk factors that increase the risk of CVD and Type 2 diabetes mellitus [38].

The exclusion criteria were smoking, malabsorption syndromes, untreated thyroid diseases, impaired kidney function, myocardial failure, insulin‐treated diabetes mellitus, chronic inflammatory diseases, cancer, alcohol or drug abuse, epilepsy, anemia, immunosuppression, long‐term intake of certain supplements (especially marine n‐3 FAs and vitamin E), and participation in another intervention study within the last 30 days.

Figure 1 shows a flowchart of the participants from the first screening to the final analysis. This study was conducted in accordance with the Declaration of Helsinki, and the study protocol was approved by the ethics committee of the Medical Faculty of the Rheinische Friedrich Wilhelms‐University of Bonn (Germany) under the identifier 420/21. All participants were informed in detail about the procedures and provided written informed consent. The study was registered in ClinicalTrials.gov (https://clinicaltrials.gov/) under the identifier NCT05208346.

### 2.2 Study Design

Before the first study day, the meal order for each subject was randomized via Williams design by the cooperating biometrician. Participants were not informed about the order of the four meals (Table 1) they received. Separated by wash‐out phases of about 14 days, the participants attended four study visits on which they received one of four different test meals. Each treatment condition lasted 6.0 h, from morning until afternoon. The meals were provided in the morning after a 10‐h overnight fast. Venous blood samples were collected before breakfast (0 h) and 0.5, 1.0, 1.5, 2.0, 3.0, 4.0, 5.0, and 6.0 h after meal ingestion. Carotid‐femoral pulse wave velocity (PWV_c‐f_) and augmentation index (AIx) were determined in the fasting condition (0 h) and at 2.0, 4.0, and 6.0 h postprandially.

**TABLE 1:** Energy content and nutrient composition of the four test meals.

	Canola oil‐containing HFM	Coconut oil‐containing HFM	Canola oil‐containing LFM	Coconut oil‐containing LFM
Energy (kJ)	4187	4202	4192	4200
Carbohydrates (g)	85.5	85.5	144.1	144.1
Carbohydrates (EN%)	35	35	59	59
Mono‐ and disaccharides (g)	39.9	39.9	73.2	73.2
Polysaccharides (g)	45.3	45.3	68.1	68.1
Ratio of polysaccharides to mono‐ and disaccharides	1.14	1.14	0.93	0.93
Dietary fiber (g)	6.6	6.6	11.2	11.2
Protein (g)	26.6	26.6	26.6	26.6
Protein (EN%)	11	11	11	11
Total fat (g)	60.9	60.5	33.6	33.4
Total fat (EN%)	54	54	30	30
SFAs (g)	8.2	45.7	5.9	24.6
SFAs (EN%)	7.3	40.2	5.2	21.7
Lauric acid (12:0) (EN%)	0.2	21.1	0.2	10.7
Myristic acid (14:0) (EN%)	0.5	8.8	0.6	4.8
Palmitic acid (16:0) (EN%)	4.6	6.8	3.0	4.0
MUFAs (g)	35.5	7.0	18.4	4.2
MUFAs (EN%)	31.4	6.2	16.2	3.7
Oleic acid (18:1n‐9) (EN%)	34.2	6.5	17.6	3.8
PUFAs (g)	15.8	2.5	8.4	1.8
PUFAs (EN%)	14.0	2.2	7.4	1.6
Linoleic acid (18:2n‐6)	10.9	2.2	5.8	1.4
α‐Linolenic acid (18:3n‐3) (EN%)	4.8	0.3	2.6	0.3
Cholesterol (mg)	210	210	15	15
Vitamin E^a^ (mg)	14.5	2.5	8.2	2.2
Vitamin C (mg)	43.6	43.6	60.0	60.0

Abbreviations: EN%, energy percentage; HFM, high‐fat meal; LFM, low‐fat meal.

^a^ α‐Tocopherol equivalents.

Throughout the entire study period, participants were instructed to maintain their habitual diet, level of physical activity, lifestyle, and weight. Participants were asked to standardize their dinner and to refrain from alcohol consumption and intensive physical activity on the day before each study visit. Participants taking antihypertensive agents (*n* = 20), lipid‐lowering drugs (*n* = 11), inhibitors of platelet aggregation (*n* = 9), thyroid therapy (*n* = 7), and antidiabetic medication (*n* = 3) were instructed to continue taking their medication without changes.

### 2.3 Test Meal Composition

Four isoenergetic (∼4200 kJ) and isonitrogenous (26.6 g protein) mixed meals (challenges) with different fat amounts and FA composition were administered on separate occasions. Each meal contained 25 or 50 g (here referred to as low‐fat meals [LFMs] or high‐fat meals [HFMs]) canola oil (Brölio canola oil; Brökelmann + Co, Hamm, Germany; rich in unsaturated FAs) or coconut oil (virgin cold pressed coconut oil; Schneekoppe, Buchholz/Nordheide, Germany; rich in SFAs). Table 2 provides an overview of the analyzed FA composition of the used plant oils and their analyzed α‐ and γ‐tocopherol content. The main FAs in canola oil were oleic acid (62.3%), linoleic acid (19.2%), and α‐linolenic acid (7.2%). Coconut oil consisted mainly of lauric acid (52.5%), myristic acid (20.1%), palmitic acid (8.4%), and capric acid (5.8%). Although 30 mg α‐tocopherol and 40 mg γ‐tocopherol were present in 100 g canola oil, neither was detectable in coconut oil.

**TABLE 2:** Fatty acid composition and α‐ and γ‐tocopherol content of the test oils.

Component	Canola oil	Coconut oil
Fatty acids	% of total fatty acids	
Caprylic acid (8:0)	nd	4.68
Capric acid (10:0)	0.01	5.82
Lauric acid (12:0)	0.01	52.53
Myristic acid (14:0)	0.05	20.06
Palmitic acid (16:0)	4.38	8.42
Stearic acid (18:0)	1.46	3.14
Oleic acid (18:1n‐9)	62.27	4.36
Linoleic acid (18:2n‐6)	19.15	0.71
α‐Linolenic acid (18:3n‐3)	7.24	nd

Tocopherols	mg/100 g	
α‐Tocopherol	30	nd
γ‐Tocopherol	40	nd

Abbreviation: nd, not detectable.

Besides the test oils, the main components of the meals were a homemade vegetable soup and other commercially available foods such as baguettes, yoghurt, jam, and fruit juice. Table 1 presents the nutrient compositions of the four test meals (canola oil‐containing HFM, canola oil‐containing LFM, coconut oil‐containing HFM, and coconut oil‐containing LFM). All meals were specifically designed for the present study, and their energy content and nutrient composition were calculated using the computer‐based nutrient calculation program EBISpro (University of Hohenheim, Stuttgart, Germany), based on the German Nutrient Database Bundeslebensmittelschlüssel (Max Rubner‐Institut, Karlsruhe, Germany). The meals were prepared by study personnel at the study site on the morning of each intervention day according to a standardized protocol, which involved weighing the food to the exact gram. The participants were requested to completely ingest the meal within 20 min under the supervision of study personnel.

### 2.4 Measurements

#### 2.4.1 Anthropometrics

Weight and height were measured using a stadiometer with an integrated scale (seca 704; Seca, Hamburg, Germany) to the nearest 0.05 kg and 0.1 cm, respectively. Waist and hip circumferences were measured in duplicate with the participants in an upright position using a flexible tape to an accuracy of 0.1 cm. Waist circumference was measured midway between the lowest rib and the iliac crest while participants were breathing calmly. Hip circumference was measured at the level of the maximum circumference above the pubic bone. Body composition (fat and fat‐free mass) was determined by air‐displacement plethysmography using the BOD POD body composition system (Cosmed, Rome, Italy) according to the instruction manual.

#### 2.4.2 BP and Heart Rate

Systolic and diastolic BP as well as heart rate were determined using an automatic BP measurement device (Boso Carat Professional; Bosch + Sohn GmbH, Jungingen, Germany) in a sitting position. The measurements were performed in duplicate under standardized conditions and according to international guidelines [39].

#### 2.4.3 Blood Sample Processing and Analysis

Fasting and postprandial blood samples were collected via an indwelling venous cannula (Vasofix Safety; B. Braun Melsungen AG, Melsungen, Germany) and drawn into tubes containing EDTA, fluoride, or a coagulation activator (SARSTEDT AG & Co. KG, Nümbrecht, Germany). Plasma and serum were obtained by centrifugation at 3000 × *g* for 15 min at 8°C. Screening blood parameters as well as TGs, total cholesterol, LDL cholesterol, HDL cholesterol, insulin, and glucose on the study days were assayed within 4 h after blood draw. Plasma and serum aliquots for quantification of nonesterified FAs (NEFAs), IL‐6, and α‐ and γ‐tocopherol, as well as for determination of trolox equivalent antioxidative capacity (TEAC) and FA profiles, were immediately frozen in cryovials and stored at −80°C until analysis.

Serum TGs, total cholesterol, LDL cholesterol, and HDL cholesterol were analyzed by fully automated photometric methods and the plasma glucose by the hexokinase method on a cobas c702 analyzer (Roche Diagnostics, Mannheim, Germany). Serum insulin concentrations were analyzed by fully automated electrochemiluminescence immunoassays (ECLIA, Elecsys tests) on a cobas e801 analyzer (Roche Diagnostics). Homeostasis model assessment for insulin resistance (HOMA‐IR) was calculated using the following formula: (insulin concentration [mU/L] × glucose concentration [mg/dL])/405. The insulin–glucose ratio was determined by dividing the insulin concentration (pmol/L) by the glucose concentration (mmol/L).

Serum CRP concentrations were measured using a turbidimetric immunoassay (cobas c702, Roche Diagnostics). Blood count parameters were analyzed by fluorescence flow cytometry, the resistance measurement technique, and photometry using a hematology analyzer (Sysmex XN9000 and Sysmex XN1000; Sysmex, Kobe, Japan). Serum gamma‐glutamyl transferase, alanine transaminase, aspartate transaminase, lipase, total bilirubin, and creatinine were measured by fully automated photometric methods (cobas c702, Roche Diagnostics).

An in vitro enzymatic colorimetric method assay (NEFA‐HR(2); FUJIFILM Wako Chemicals Europe GmbH, Neuss, Germany) was used to measure the serum NEFA concentration in duplicate. The plasma concentration of IL‐6 was analyzed in duplicate using a commercially available ELISA (R&D Systems, Minneapolis, USA). Plasma concentrations of α‐ and γ‐tocopherol were determined via HPLC as described previously [40]. Serum FA profiles were determined in duplicate by gas chromatography as described previously [41]. The plasma total antioxidant capacity was determined using the TEAC method developed by Miller et al. [42].

#### 2.4.4 Parameters of Arterial Stiffness

PWV_c‐f_ and AIx were determined using a Vicorder device (SMT Medical Technology GmbH, Würzburg, Germany). Before starting the measurement, the participants rested in a quiet and well‐tempered examination room for 2 min in a supine position on an examination bed with the headboard angled at 30°. In the first step, one of the inflatable cuffs was attached centrally on the upper arm to conduct pulse wave analysis, which includes the determination of AIx. After an initial BP measurement, pulse wave analysis was performed. In the second step, the inflatable cuff was attached around the upper thigh to determine PWV_c‐f_. After measuring the distance between the cuff and the jugulum using a special compass to adjust for the convexity of the torso, a neck cuff was tightly attached at the level of the carotid artery. The mean value of two measurements was used for statistical analysis of the two arterial stiffness markers.

#### 2.4.5 Self‐Reported Physical Activity and Dietary Intake of Energy and Nutrients

Participants were instructed to complete a 1‐day food diary and physical activity log on the day before each study visit to identify possible variations in food intake and physical activity. The food diaries were analyzed using the computer‐based nutrient calculation program EBISpro (University of Hohenheim, Stuttgart, Germany). Each subject additionally completed a 3‐day food diary once before the start of the study to assess their habitual diet.

### 2.5 Statistical Analyses

The postprandial TG concentration was used as the primary outcome to determine the sample size. The calculation was based on data from previous postprandial studies of the study group with comparable study designs [43, 44] and anticipated postprandial changes of the TG concentration in the four treatment conditions (one‐factor analysis of variance [ANOVA], *p* < 0.05). The calculation indicated that a sample size of 24 participants allowed detection of a difference of 0.14 mmol/L in the TG concentration with a power of 80%, assuming an SD of 0.23 mmol/L. Considering possible drop‐outs, 30 participants were included in the study. Drop‐outs were to be replaced using the same sequence of meals for the newly enrolled subject.

All statistical analyses were performed using the IBM SPSS statistical software package, version 28 (IBM, Armonk, NY, USA). Data were analyzed according to a prespecified analysis plan, which was finalized before outcome data were available. The significance level was set at 0.05. Baseline characteristics of men and women were compared using the unpaired Student's *t* test. Data of the 1‐day food diaries and physical activity logs were compared between the four pretreatment days using a one‐factor ANOVA.

The effects of the interventions (fat type: coconut oil and canola oil; fat amount: low‐fat and high‐fat), time points (fasting and 0.5, 1.0, 1.5, 2.0, 3.0, 4.0, 5.0, and 6.0 h postprandially), and their interactions (fat amount × fat type, fat amount × time, fat type × time, and fat amount × fat type × time) on all postprandial parameters were tested using a linear mixed model with repeated measures; time within a subject and period was treated as repeated effect. The interventions, time points, and their interactions were set as fixed factors, as were the visit, but without interaction with the previous ones. The subject identifier was included as a random variable, and the fasting value was included as a covariate. When there was no significant interaction between the factors, the linear mixed model was repeated without the respective interaction term. To assess the adequacy of the statistical tests, the residuals resulting from the analysis were assessed for a normal distribution. When there were non‐normally distributed residuals, the linear mixed model was rerun with log‐transformed data.

Additionally, for each parameter, the incremental area under the curve (iAUC) was calculated using the linear trapezoidal rule and analyzed using the linear mixed model. Here, fat type, fat amount, and their interaction as well as the visit were set as fixed factors. The subject identifier was included as a random variable.

## 3 Results

### 3.1 Baseline Characteristics

In total, 30 participants were included in this study. Table 3 provides an overview of the baseline characteristics of the 29 participants (age, 70.0 ± 5.3 years; BMI, 30.2 ± 2.6 kg/m^2^) who completed all study days. One woman dropped out during the intervention period due to difficulties in fulfilling the requirements of the study protocol. Height (*p* < 0.001), weight (*p* = 0.008), waist circumference (*p* = 0.045), the waist‐to‐hip ratio (*p* < 0.001), and fat mass (*p* < 0.001) significantly differed between men and women.

**TABLE 3:** Baseline characteristics of subjects who completed the study.

	Total (*n *= 29)	Men (*n *= 18)	Women (*n *= 11)	*p* value
Age (year)	70.0 ± 5.3	69.0 ± 5.4	71.7 ± 4.8	0.179
Height (cm)	171.6 ± 8.3	176.6 ± 5.0	163.5 ± 5.7	<0.001
Weight (kg)	89.1 ± 10.1	92.8 ± 8.8	83.0 ± 9.3	0.008
BMI (kg/m^2^)	30.2 ± 2.6	29.8 ± 2.5	31.0 ± 2.7	0.217
Waist circumference (cm)	107.1 ± 7.9	109.4 ± 7.2	103.4 ± 7.9	0.045
Hip circumference (cm)	107.6 ± 6.6	105.9 ± 5.5	110.5 ± 7.6	0.066
Waist‐to‐hip ratio	0.997 ± 0.069	1.034 ± 0.051	0.937 ± 0.048	<0.001
Fat mass (%)	39.2 ± 8.3	33.7 ± 4.3	48.3 ± 3.9	<0.001
Systolic BP (mmHg)	150.7 ± 18.3	154.9 ± 14.7	143.8 ± 22.0	0.113
Diastolic BP (mmHg)	87.4 ± 9.7	88.9 ± 11.4	84.9 ± 5.7	0.284
Pulse (min^−1^)	64.7 ± 8.8	65.4 ± 9.8	63.5 ± 7.0	0.563
Serum triglycerides (mmol/L)	1.83 ± 0.77	1.89 ± 0.87	1.74 ± 0.60	0.633
Serum total cholesterol (mmol/L)	5.58 ± 1.22	5.60 ± 1.27	5.55 ± 1.20	0.907
Serum HDL cholesterol (mmol/L)	1.45 ± 0.47	1.37 ± 0.53	1.58 ± 0.34	0.250
Serum LDL cholesterol (mmol/L)	3.51 ± 1.02	3.60 ± 1.02	3.36 ± 1.06	0.551
Plasma glucose (mmol/L)	5.88 ± 1.05	5.97 ± 1.20	5.74 ± 0.78	0.572
Serum CRP (mg/L)	2.05 ± 1.52	1.82 ± 1.33	2.39 ± 1.78	0.344

Data are shown as mean ± SD. All blood parameters were measured at the screening visit in fasting samples. Values of men and women were compared using the unpaired Student's *t* test.

Abbreviations: BP, blood pressure; CRP, C‐reactive protein.

### 3.2 Dietary Intake and Physical Activity on Pretreatment Days

Based on 1‐day food diaries and physical activity logs, participants maintained their habitual dietary pattern and level of physical activity throughout the entire study phase. Total energy and macronutrient intake and the physical activity level did not significantly differ between the four pretreatment days. Additionally, habitual energy and macronutrient intake, assessed by 3‐day food diaries, did not differ from the intake on pretreatment days (data not shown).

### 3.3 Analysis of Fasting and Postprandial Parameters in Plasma/Serum

#### 3.3.1 Serum Lipids, Lipoproteins, and NEFAs

**FIGURE 2:**
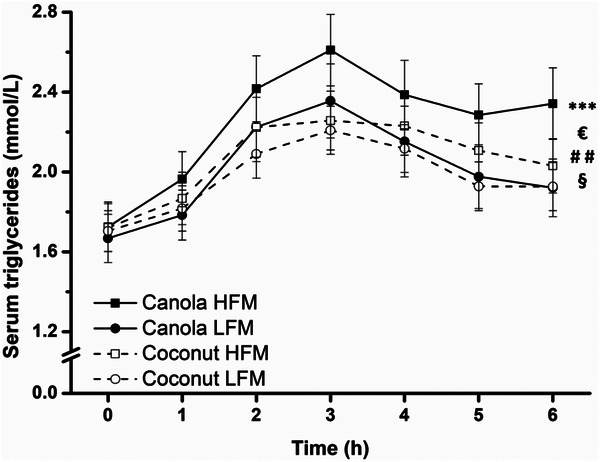
Fasting and postprandial concentrations of serum triglycerides in response to test meals in adults with increased cardiometabolic risk. Canola oil consumption resulted in a higher triglyceride concentration than coconut oil consumption. Data are shown as mean ± SEM (*n* = 29). A linear mixed model with repeated measures was used to test for effects of interventions, time points, and their interactions. ***p < 0.001 for fixed factor time, €*p* < 0.05 for fixed factor fat type, ##*p* < 0.01 for fixed factor fat amount, §*p* < 0.05 for fat type × time interaction. Abbreviations: HFM, high‐fat meal; LFM, low‐fat meal.

In response to all meals, the TG concentration increased up to 3.0 h postprandially and then decreased (time effect *p* < 0.001) (Figure 2). At the end of the observation period, the TG concentration was still above the preprandial level for all meals. A significant fat type effect (*p* = 0.013) and a significant fat type × time interaction (*p* = 0.035) were observed, meaning that canola oil provoked stronger lipemia than coconut oil. Additionally, HFMs provoked more extensive lipemia, indicated by a significant effect of fat amount (*p* = 0.004). iAUC data confirmed the effect of fat type (canola vs. coconut 47.9 ± 15.2 mmol/L × min, *p* = 0.002) and fat amount (HFM vs. LFM 45.2 ± 15.2 mmol/L × min, *p* = 0.004) on the postprandial TG concentration (Table 4, Figure S1).

**TABLE 4:** Postprandial responses shown by the incremental area under the curve for blood parameters of participants with a risk phenotype for cardiometabolic diseases.

	Canola oil‐containing HFM	Canola oil‐containing LFM	Coconut oil‐containing HFM	Coconut oil‐containing LFM	*p* value (fat amount)	*p* value (fat type)
Lipid and glucose metabolism						
Triglyceride iAUC (mmol/L × min)	198.4 ± 18.9	136.7 ± 22.0	133.8 ± 13.8	105.0 ± 15.1	0.004	0.002
NEFA iAUC (mmol/L × min)	−62.9 ± 6.3	−90.8 ± 10.0	−67.5 ± 13.8	−89.0 ± 9.6	0.002	0.835
Total cholesterol iAUC (mmol/L × min)	−49.5 ± 10.9	−64.7 ± 11.4	−36.8 ± 9.1	−73.3 ± 15.3	0.019	0.870
HDL cholesterol iAUC (mmol/L × min)	−17.3 ± 3.3	−15.2 ± 3.4	−14.6 ± 3.3	−18.0 ± 3.3	0.820	0.970
LDL cholesterol iAUC (mmol/L × min)	−53.9 ± 8.7	−49.5 ± 9.6	−33.4 ± 6.00	−53.8 ± 6.8	0.252	0.261
Glucose iAUC (mmol/L × min)	60.7 ± 33.5	173.6 ± 44.7	29.1 ± 43.5	164.2 ± 53.7	<0.001	0.617
Insulin iAUC (nmol/L × min)	100.6 ± 16.4	146.5 ± 26.7	102.3 ± 18.1	144.9 ± 22.5	<0.001	0.990

Inflammation and oxidation						
IL‐6 iAUC (pg/mL × min)	355.6 ± 91.8	280.2 ± 59.2	178.7 ± 51.4	183.9 ± 61.9	0.574	0.047
TEAC iAUC (mmol trolox equivalent/L × min)	1.9 ± 4.5	−16.8 ± 6.7	−10.1 ± 5.9	0.1 ± 7.4	0.468	0.713
α‐Tocopherol iAUC (µg/mL × min)	−124.4 ± 54.4	−228.0 ± 49.5	−114.5 ± 44.6	−171.5 ± 108.6	0.195	0.617
γ‐Tocopherol iAUC (µg/mL × min)	24.4 ± 6.6	1.8 ± 6.5	−18.3 ± 3.5	−21.3 ± 8.6	0.020	<0.001

Data are shown as mean ± SEM (*n *= 29). A linear mixed model was used to test for effects of interventions, time points, and their interactions.

Abbreviations: HFM, high‐fat meal; iAUC, incremental area under the curve; LFM, low‐fat meal; NEFA, nonesterified fatty acid; TEAC, trolox equivalent antioxidative capacity.

Concentrations of HDL, LDL, and total cholesterol decreased postprandially, resulting in a significant time effect (all *p* < 0.001) (Figure S2). A significant fat type × fat amount interaction was observed for LDL cholesterol in time‐course profiles (*p* = 0.032), but not in iAUC data. For total cholesterol, a significant fat amount effect (*p* = 0.014) and a significant fat amount × time interaction (*p* = 0.007) were observed. The effect of fat amount on total cholesterol was also observed in iAUC data (LFM vs. HFM −25.8 ± 10.8 mmol/L × min, *p* = 0.019), indicating total cholesterol decreased more after LFMs than after HFMs (Table 4).

**FIGURE 3:**
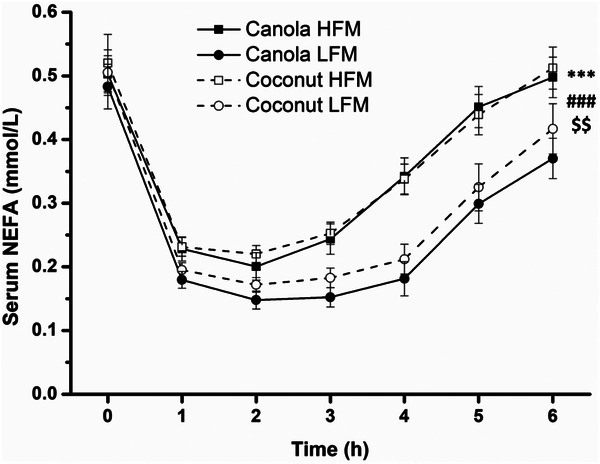
Fasting and postprandial concentrations of serum nonesterified fatty acids in response to test meals in adults with increased cardiometabolic risk. Low‐fat meal consumption resulted in a lower NEFA concentration than high‐fat meal consumption. Data are shown as mean ± SEM (*n* = 29). A linear mixed model with repeated measures was used to test for effects of interventions, time points, and their interactions. ***p < 0.001 for fixed factor time, ###*p* < 0.001 for fixed factor fat amount, $$*p* < 0.01 for fat amount × time interaction. Abbreviations: HFM, high‐fat meal; LFM, low‐fat meal; NEFA, nonesterified fatty acid.

After all meals, the NEFA concentration decreased up to 2.0 h postprandially and then increased (time effect *p* < 0.001) (Figure 3). Throughout the entire postprandial period, NEFA concentrations were significantly lower after LFMs, resulting in a significant effect of fat amount (*p* < 0.001) and a significant fat amount × time interaction (*p* = 0.007). The effect of fat amount on the NEFA concentration was confirmed by analysis of iAUC data (LFM vs. HFM −24.6 ± 7.5 mmol/L × min, *p* = 0.002) (Table 4, Figure S3).

#### 3.3.2 Serum FA Profiles

Analysis of FA time‐course profiles showed a significant time effect for lauric, myristic, oleic, and α‐linolenic acid (all *p* < 0.001) (Figure S4). Concentrations of lauric and myristic acid were higher after coconut oil consumption, while concentrations of oleic, linoleic, and α‐linolenic acid were higher after canola oil consumption. This was reflected in a significant fat type effect and a significant fat type × time interaction for lauric, myristic, oleic, and α‐linolenic acid (all *p* < 0.001). For linoleic acid, only a significant fat type effect was observed (*p* = 0.005) (Figure S4).

#### 3.3.3 Plasma Glucose, Serum Insulin, HOMA‐IR, and the Insulin–Glucose Ratio

**FIGURE 4:**
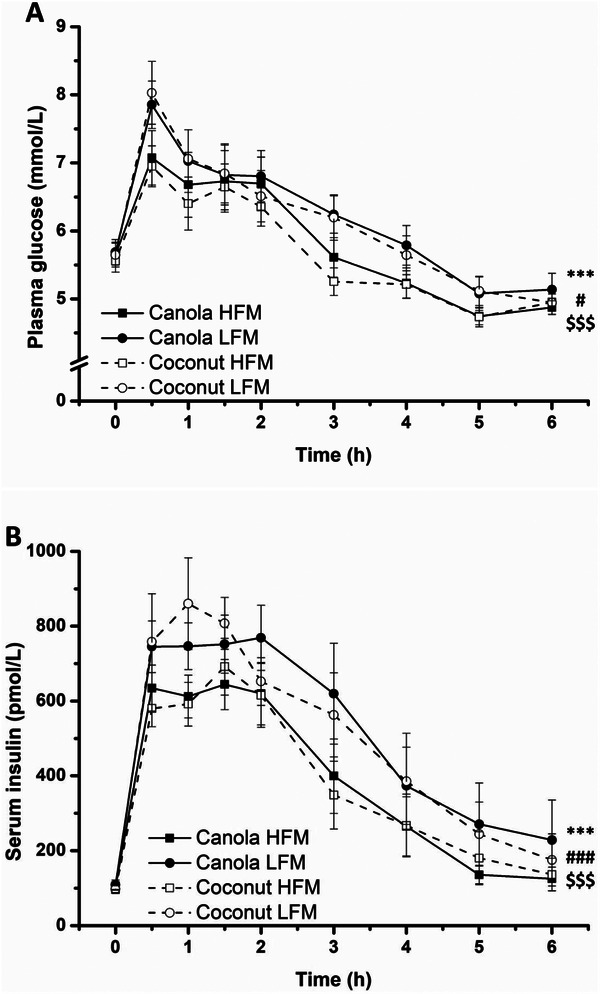
Fasting and postprandial concentrations of plasma glucose (A) and serum insulin (B) in response to test meals in adults with increased cardiometabolic risk. Low‐fat meal consumption resulted in higher glucose and insulin concentrations than high‐fat meal consumption. Data are shown as mean ± SEM (*n* = 29). A linear mixed model with repeated measures was used to test for effects of interventions, time points, and their interactions. ***p < 0.001 for fixed factor time, #*p* < 0.05 for fixed factor fat amount, ###*p* < 0.001 for fixed factor fat amount, $$$*p* < 0.001 for fat amount × time interaction. Abbreviations: HFM, high‐fat meal; LFM, low‐fat meal.

In response to all meals, the glucose concentration increased from baseline, peaked at 0.5 h postprandially, and gradually decreased to below the baseline at 6.0 h postprandially (time effect *p* < 0.001). Likewise, the insulin concentration increased, plateaued between 0.5 and 2.0 h postprandially, and subsequently decreased (time effect *p* < 0.001) (Figure 4). LFMs provoked stronger glycemic and insulinemic responses, reflected in significant fat amount effects (*p* = 0.016 and *p* < 0.001) and fat amount × time interactions (both *p* < 0.001). Similar effects as those in the glucose and insulin time‐course profiles were observed for HOMA‐IR and the insulin–glucose ratio (Figure S5). Analysis of iAUC data confirmed the significant effect of fat amount on glucose and insulin (LFM vs. HFM 127.9 ± 31.8 mmol/L × min, *p* < 0.001; LFM vs. HFM 43.7 ± 6.7 nmol/L × min, *p* < 0.001) (Table 4, Figure S6).

#### 3.3.4 Plasma IL‐6, α‐ and y‐Tocopherol, and TEAC

**FIGURE 5:**
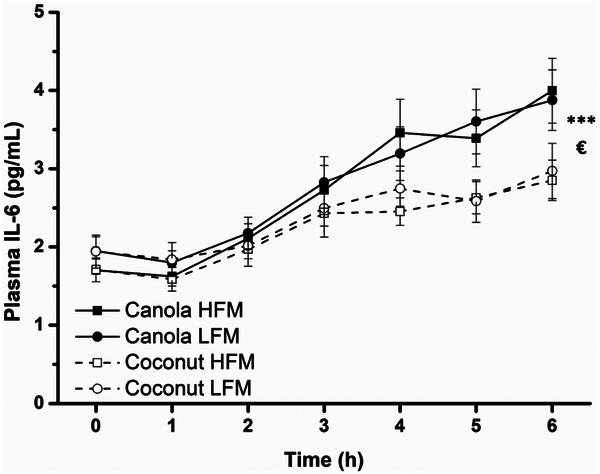
Fasting and postprandial concentrations of plasma IL‐6 in response to test meals in adults with increased cardiometabolic risk. Canola oil consumption resulted in a higher IL‐6 concentration than coconut oil consumption. Data are shown as mean ± SEM (*n* = 29). A linear mixed model with repeated measures was used to test for effects of interventions, time points, and their interactions. ***p < 0.001 for fixed factor time, €*p* < 0.05 for fixed factor fat type. Abbreviations: HFM, high‐fat meal; LFM, low‐fat meal.

The concentration of IL‐6 significantly increased after all meals (time effect *p* < 0.001) and was higher after canola oil consumption (fat type effect *p* = 0.025) (Figure 5). This significant fat type effect was confirmed by iAUC data (canola vs. coconut 131.1 ± 65.0 pg/mL × min, *p* = 0.047) (Table 4, Figure S7). The iAUC data of TEAC showed a significant fat type × fat amount interaction (*p* = 0.023), which was not observed in time‐course profiles (data not shown). There were no effects of fat type or fat amount on TEAC (Table 4).

**FIGURE 6:**
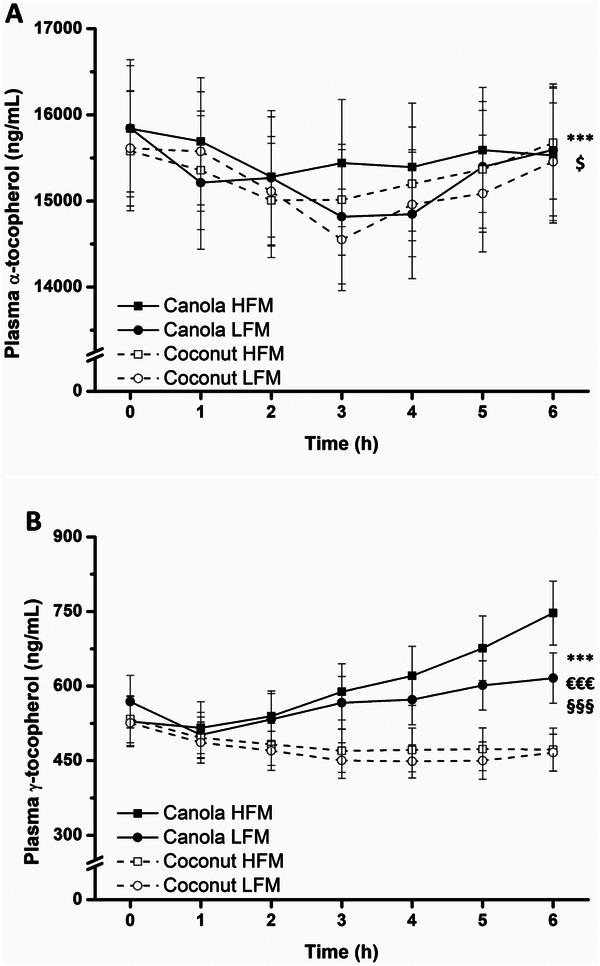
Fasting and postprandial concentrations of plasma α‐tocopherol (A) and plasma γ‐tocopherol (B) in response to test meals in adults with increased cardiometabolic risk. Canola oil consumption resulted in a higher γ‐tocopherol concentration than coconut oil consumption. Data are shown as mean ± SEM (*n* = 29). A linear mixed model with repeated measures was used to test for effects of interventions, time points, and their interactions. ***p < 0.001 for fixed factor time, €€€*p* < 0.001 for fixed factor fat type, §§§*p* < 0.001 for fat type × time interaction, $*p* < 0.05 for fat amount × time interaction. Abbreviations: HFM, high‐fat meal; LFM, low‐fat meal.

Time‐course profiles of α‐tocopherol showed a significant time effect (*p* < 0.001) and a significant fat amount × time interaction (*p* = 0.039) (Figure 6A). In addition to a significant time effect (*p* < 0.001), concentrations of γ‐tocopherol were significantly higher after canola oil consumption, indicated by a significant fat type effect and fat type × time interaction (both *p* < 0.001) (Figure 6B). Although iAUC data revealed no effect of fat amount or fat type on α‐tocopherol, both factors significantly influenced the iAUC of γ‐tocopherol (HFM vs. LFM 13.2 ± 5.6 µg/mL × min, *p* = 0.020; canola vs. coconut 32.9 ± 5.5 µg/mL × min, *p* < 0.001) (Table 4, Figure S8).

### 3.4 Parameters of Arterial Stiffness

PWV_c‐f_ and AIx values postprandially decreased until 2.0 h and then increased. At the end of the observation period, AIx values were below the baseline. A significant time effect was observed for both PWV_c‐f_ and AIx (both *p* < 0.001). No effect of fat amount or fat type was detected in the time‐course profiles of PWV_c‐f_ and AIx (Figure S9).

## 4 Discussion

This randomized controlled postprandial trial aimed to investigate the acute effects of four mixed meals enriched with 25 or 50 g canola or coconut oil on the metabolic response and parameters of arterial stiffness in overweight and obese older adults with a risk phenotype for cardiometabolic diseases. To the best of our knowledge, this is the first randomized controlled trial on canola and coconut oil that analyzed a wide range of parameters addressing the metabolic response and arterial stiffness as well as the postprandial FA profile.

Our major finding was that consumption of canola oil resulted in a stronger TG response than consumption of coconut oil. The strong increase of the TG concentration observed after canola oil consumption may be attributed to the fact that canola oil consists almost entirely of FAs with a chain length of at least 16 carbon atoms, which are incorporated into chylomicrons and released into the systemic circulation via the lymphatic system [45, 46]. The lower TG response after coconut oil consumption indicates that specific FAs, mainly caprylic and capric acid (which together account for approximately 10.5% of the total FA content of coconut oil), were directly transported via the portal vein to the liver. The marked increase in the TG concentration after coconut oil consumption and the high concentrations of lauric acid found in postprandial serum supports the assumption that lauric acid was metabolized as a long‐chain FA. The transport of lauric acid via chylomicrons was additionally confirmed by the similar time‐course profiles of lauric acid and long‐chain FAs analyzed in blood serum (e.g., oleic and α‐linolenic acid).

Another reason for the observed effect of fat type on the TG concentration may be possible differences in the degradation behavior of chylomicrons with varying compositions because it has been suggested that FA classes influence the composition of TG‐rich lipoproteins [47]. With regard to the present trial, this means that chylomicrons rich in lauric acid would have been degraded faster than chylomicrons rich in oleic acid. Future studies regarding the influence of different dietary fats on postprandial lipemia should measure the concentration and composition of chylomicrons to investigate the lipid response to test meals in more detail.

In addition to the differential effect of fat type on the lipemic response, we found a robust effect of fat amount on lipemia, meaning that the higher fat dose of a plant oil (HFMs, 50 g test oil) triggered a stronger TG response than the corresponding meal with the lower fat dose (LFMs, 25 g test oil). This finding is consistent with the well‐established concept that the TG response gradually increases with an increasing dietary fat dose [48, 49]. The observation that the TG concentration peaked 3 h postprandially is also consistent with other trials in which fat‐containing meals were administered [43, 44, 50, 51].

In the present study, ingestion of all four meals induced glycemia and insulinemia as well as significant increases in the derived parameters HOMA‐IR and the insulin–glucose ratio. The intensive insulin secretion between 0.5 and 2.0 h postprandially caused a rapid decline of the glucose concentration to values below the baseline after it had peaked at 0.5 h after meal consumption. The more extensive glycemic and insulinemic response after LFMs can be attributed to their higher contents of carbohydrates (LFMs: 144.1 g vs. HFMs: 85.5 g) and mono‐ and disaccharides (LFMs: 73.2 g vs. HFMs: 39.9 g) than HFMs. This is consistent with a recent meta‐analysis describing the attenuating effect on acute glucose and insulin responses by exchanging carbohydrates for fats in mixed meals [52]. Concerning the test oils, the results of our trial suggest that the FA composition of a meal does not influence the postprandial concentrations of glucose and insulin. This assumption is confirmed by other acute studies that observed no differential effect of meals high in SFAs, MUFAs, or PUFAs on glucose or insulin levels [53, 54, 55, 56].

Insulin is a potent inhibitor of intracellular lipases [57]. For example, insulin reduces the activity of hormone‐sensitive lipase, which is required for complete hydrolysis of triacylglycerol and release of NEFAs [58]. Hence, the increase in the insulin concentration observed in the early postprandial phase is closely linked to the rapid decrease of the NEFA concentration within the first 2 h after meal intake. Given this inverse relationship between insulin and NEFA concentrations, it stands to reason that consumption of LFMs not only triggered a stronger insulinemic response than consumption of HFMs but also resulted in a greater decrease of NEFAs. At the end of the postprandial period, the NEFA concentration typically increases above the preprandial values [57]. It can be assumed that due to the strong decrease of the NEFA concentration in the early postprandial period and its gradual increase in the later phase, our postprandial observation period of 6 h might have been too short to capture the rebound of the NEFA concentration above postabsorptive values. A postprandial observation period of 8 h would have been useful to capture the rebounding effect of NEFAs [59].

In a comprehensive review of the inflammatory response to single HFMs, Emerson et al. [60] concluded that of five common inflammatory markers, only IL‐6 consistently increased postprandially. Accordingly, in our trial, the IL‐6 concentration significantly increased after all four test meals. This distinct time effect has also been demonstrated in two former trials of our study group [43, 44]. In addition, there is mechanistic evidence that the postprandial increase in IL‐6 results from activation of inflammatory signaling cascades, in particular increased DNA‐binding activity of the transcription factor nuclear factor kappa‐light‐chain‐enhancer of activated B cells (NF‐κB) and increased degradation of the inhibitory protein IκB‐α [61]. In the context of assessing inflammatory markers in postprandial protocols, it should be considered that the inflammatory response after meal consumption is regarded as a physiological phenomenon [62], and the body is able to successfully adapt to this meal‐induced “stress” [63]. Regarding the fact that canola oil induced a stronger IL‐6 response than coconut oil, a connection with its stronger TG response may be assumed because changes in the TG concentration are associated with changes in markers of postprandial inflammation [64, 65]. However, no association between TGs and IL‐6 in response to canola oil‐containing meals was observed in our data, neither in the correlation analysis for repeated measures (canola HFM *r* = −0.05, canola LFM *r* = −0.12) nor in the mediation analysis assessing whether the IL‐6 response to canola oil‐containing meals was mediated by TGs (indirect effect *ab* = −11.18, 95% CI [−73.37, 32.56]). The proinflammatory state after HFMs may result from leukocyte activation induced by chylomicron remnants [66]. In general, it appears that patterns and clusters of inflammatory markers may be more robust than single markers to comprehensively characterize the inflammatory response [67].

After ingestion of HFMs, we observed significant increases in serum concentration of FAs derived from oils, meaning that the concentrations of lauric and myristic acid strongly increased in response to the coconut oil‐containing HFM, while the concentrations of oleic, linoleic, and α‐linolenic acid markedly increased after the canola oil‐containing HFM. Analogous to the serum FA profile, we were able to depict the postprandial change in γ‐tocopherol. The concentration increased most after the canola oil‐containing HFM (20 mg γ‐tocopherol/50 g canola oil), followed by the canola oil‐containing LFM (10 mg γ‐tocopherol/25 g canola oil). No increment was observed after the coconut oil‐containing meals due to the fact that γ‐tocopherol could not be detected in this oil. The absence of an increase in the α‐tocopherol level during the postprandial period can be explained by a relatively high α‐tocopherol concentration at baseline, especially compared to γ‐tocopherol, and a rather low α‐tocopherol intake via the test meals.

In this study, consumption of all meals significantly decreased AIx, which is in accordance with the results of our recently published review [68]. Similarly, PWV_c‐f_ values significantly decreased postprandially regardless of the fat amount and the FA composition of the meal. Little research has been conducted on the effects of the fat amount and FA composition of mixed meals on PWV_c‐f_, and the available evidence is inconsistent [68, 69, 70]. Due to substantial deviations in the study protocols (e.g., study groups and test oils), it would be questionable to compare our data with these trials. It remains unclear whether the decreases in AIx and PWV_c‐f_ are attributable to the consumption of the test meals or to the circadian rhythm. Since both AIx and PWV_c‐f_ are largely determined by BP [71, 72], there may be a connection between the postprandial decreases in AIx and PWV_c‐f_ and the physiological decreases in systolic and diastolic BP, which occur after food intake due to the activation of the parasympathetic system [73].

### 4.1 Strengths and Limitations

The main strengths of this study are the randomized crossover design, controlled setting, and high treatment compliance. Strong deviations in the environmental conditions would have been apparent and could have been considered in the data analysis by monitoring nutrient intake and physical activity on pretreatment days. The well‐planned protocol enabled us to analyze a great variety of metabolic parameters and the vascular response. Short time intervals between blood collections allowed us to continuously monitor postprandial responses, which is beneficial with regard to the rapid changes in metabolic parameters such as glucose and insulin levels. The determination of the postprandial serum FA profile in addition to common metabolic parameters enabled us to better understand the effects of the two test oils on the TG response. Compared with previous studies [43, 44, 69, 74, 75], in this trial, we chose a prolonged observation period of 6 h to capture metabolic responses in the late phase of digestion. This trial helps to clarify the metabolic effects of tropical fats that are increasingly used in modern cuisine by analyzing the postprandial effects of coconut oil in comparison with those of canola oil. This study administered complete meals with commercially available foods instead of liquid tolerance test meals, which further enhances its practical relevance.

Despite the prolonged observation period, the TG concentration was still above the preprandial level after 6 h. A further extension of the observation period would have allowed us to describe the metabolic response in even greater detail, but would have placed too great a burden on the study participants. When interpreting the significant effects of fat amount (e.g., on glucose, insulin), it should be noted that the variation in fat content between HFMs and LFMs was accompanied by a variation in carbohydrate content, as the meals were intended to be isoenergetic and isonitrogenous. Another potential limitation of this study is that the results are of limited transferability to other populations (e.g., metabolically healthy individuals); however, especially for the vulnerable group of participants with increased cardiometabolic risk, attenuating the postprandial metabolic response by dietary FA modification might be an effective strategy to reduce the CVD risk [76]. The organization of the study did not allow double‐blinding in full, meaning that only measurements (blood drawing and analyses, measurement of arterial stiffness parameters) but not the serving of meals occurred in a double‐blinded manner. Nevertheless, we assume that the lack of full double‐blinding had no effect on our data, especially because statistical analyses were conducted without knowledge of the randomization protocol. It should be noted that our study analyzed the acute effects of canola and coconut oil on metabolism. For a further nutritional‐physiological evaluation of both oils, their long‐term effects on CVD‐related parameters should also be considered. In this context, canola oil has a beneficial effect on several cardiometabolic risk markers [5, 6], while coconut oil is associated with increased LDL cholesterol levels [1, 9, 77].

In conclusion, our results show that in adults with a risk phenotype for CVDs, both the meal fat amount and its FA composition affect cardiometabolic risk factors. Canola and coconut oil induced different metabolic responses, and the manner and strength of the effects differed depending on the parameter. For the further physiological evaluation of dietary fats, more well‐designed and highly standardized studies on the acute and longer‐term metabolic effects of plant oils used in modern cuisine are needed. Future studies should consider the following aspects in particular: postprandial period of at least 6–8 h, randomized crossover design, well‐characterized intervention (e.g., analyzed composition of test meals), further studies with different study populations (e.g., metabolically healthy individuals, risk phenotype, or risk genotype for CVDs), and measurement of a wide range of physiological and immunological parameters.

## Conflicts of Interest

The authors declare no conflicts of interest.

## Supporting information




**Supporting File 1**: mnfr70147‐supp‐0001‐SuppMat.docx.

## Data Availability

Data described in the manuscript can be made available upon request pending application and approval.
