# Supplementary material for: Postprandial Responses to Meals Enriched With Canola or Coconut Oil in Men and Women With a Risk Phenotype for Cardiometabolic Diseases: A Randomized Crossover Trial
Source: Mol Nutr Food Res. 2025 Jun 19;69(19):e70147. doi: 10.1002/mnfr.70147 (PMC12490190; doi:10.1002/mnfr.70147)
Supplement: Supplementary file 1 — Supporting File 1: mnfr70147‐supp‐0001‐SuppMat.docx. [file MNFR-69-e70147-s001.docx]

**
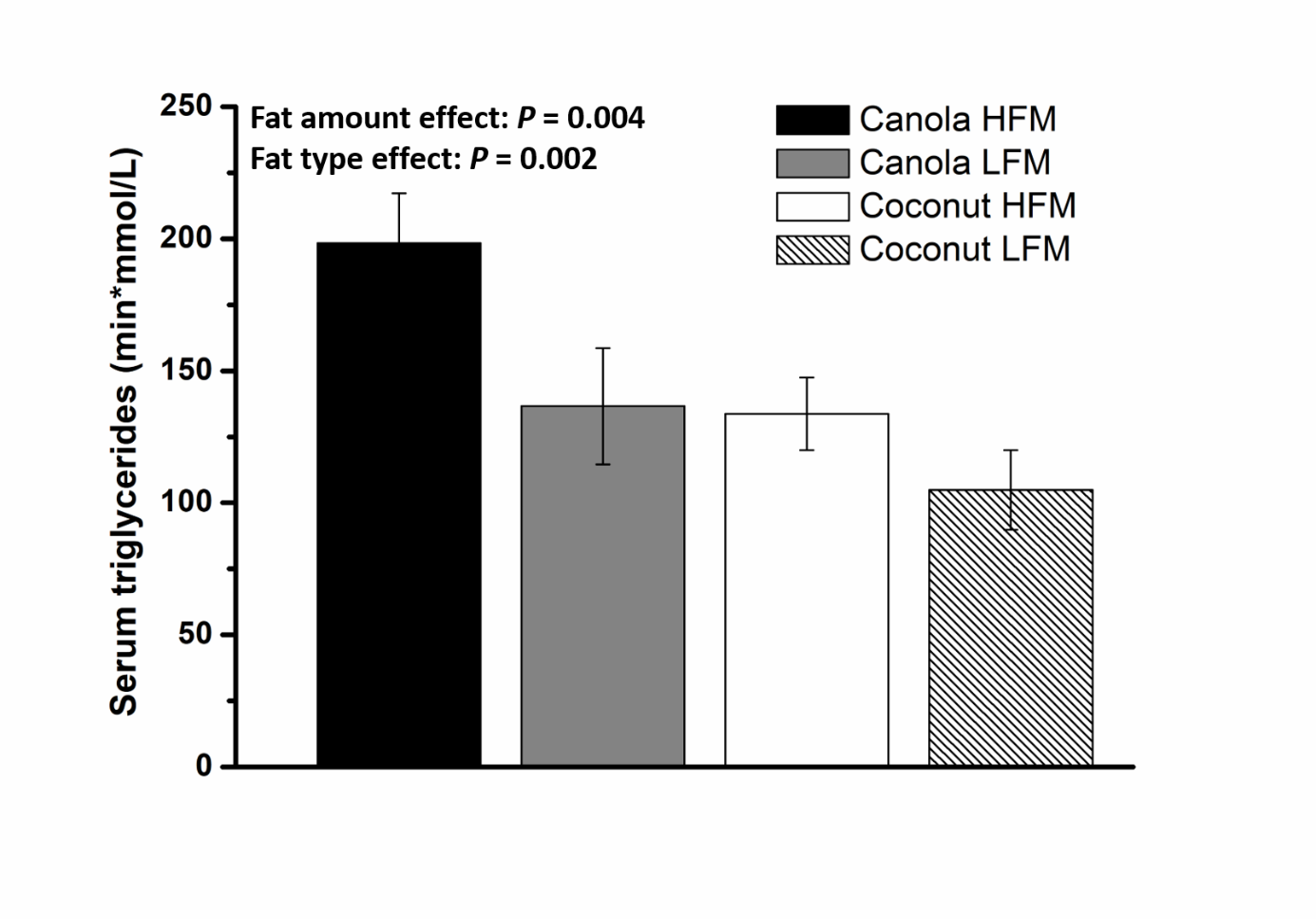
**

**SUPPLEMENTARY FIGURE 1.** Effects of test meals on postprandial concentrations of serum triglycerides shown by incremental area under the curve. Data are shown as mean ± SEM (*n* = 29). A linear mixed model was used to test for effects of interventions, time points, and their interactions. Abbreviations: HFM, high-fat meal; LFM, low-fat meal.


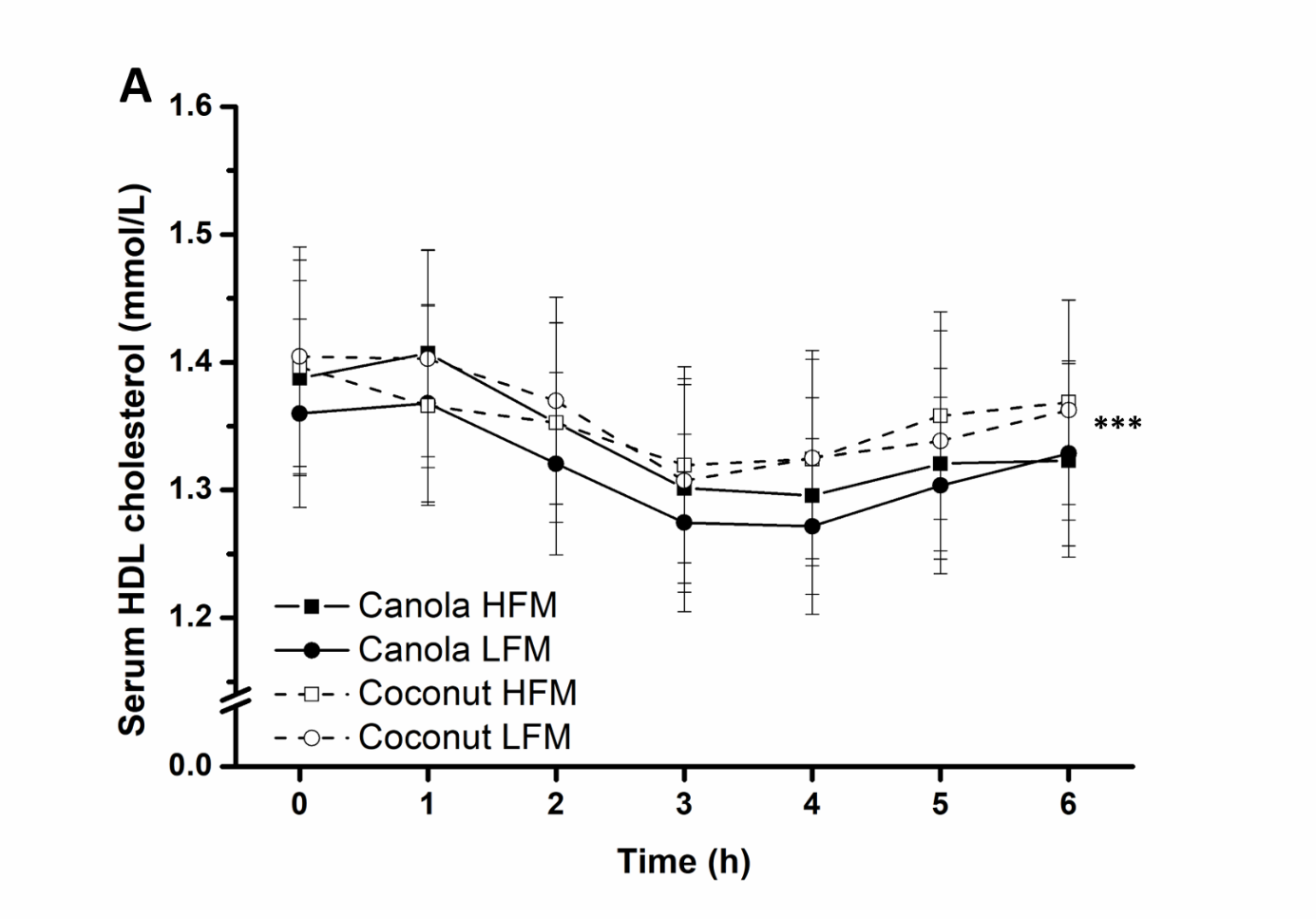


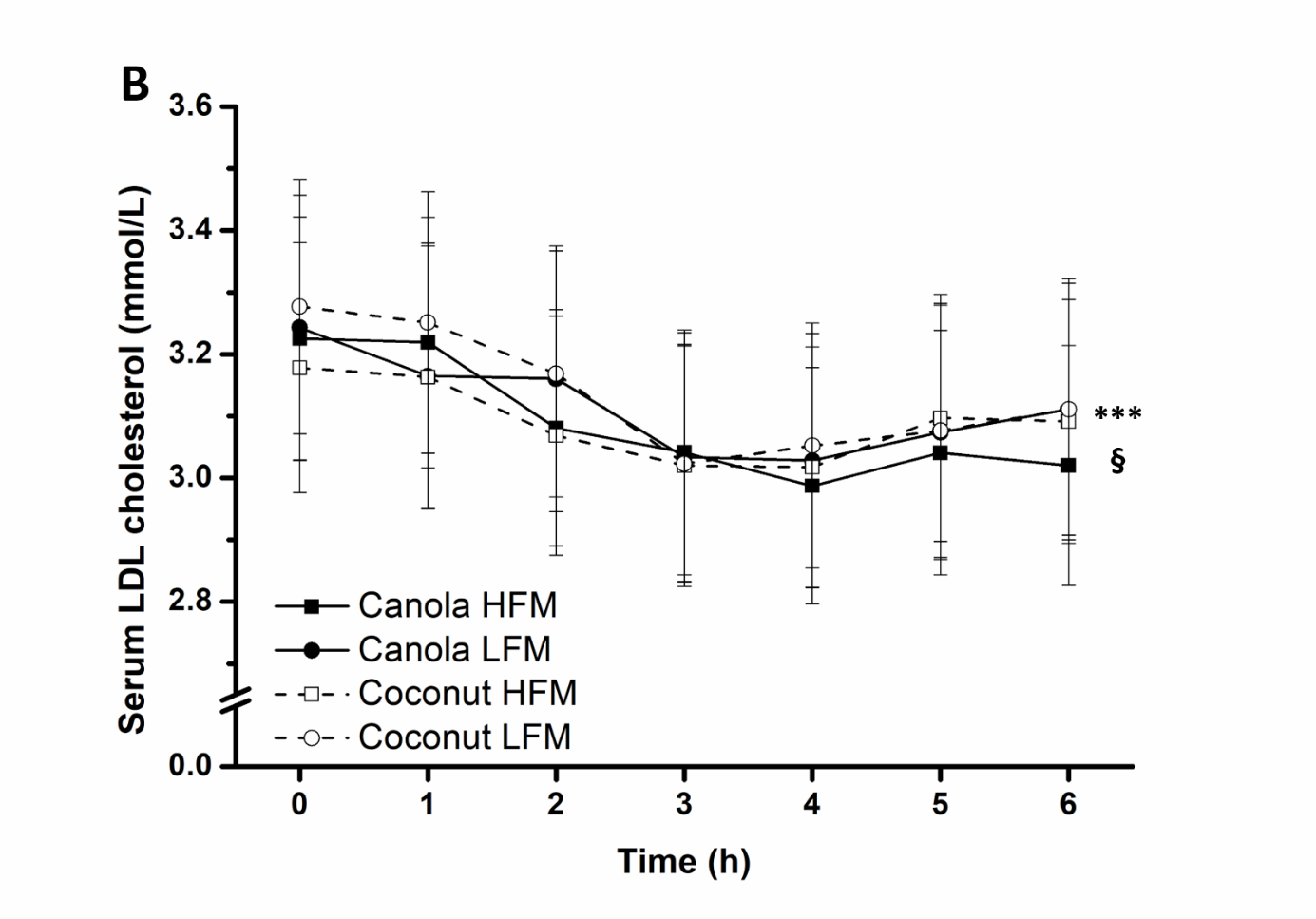


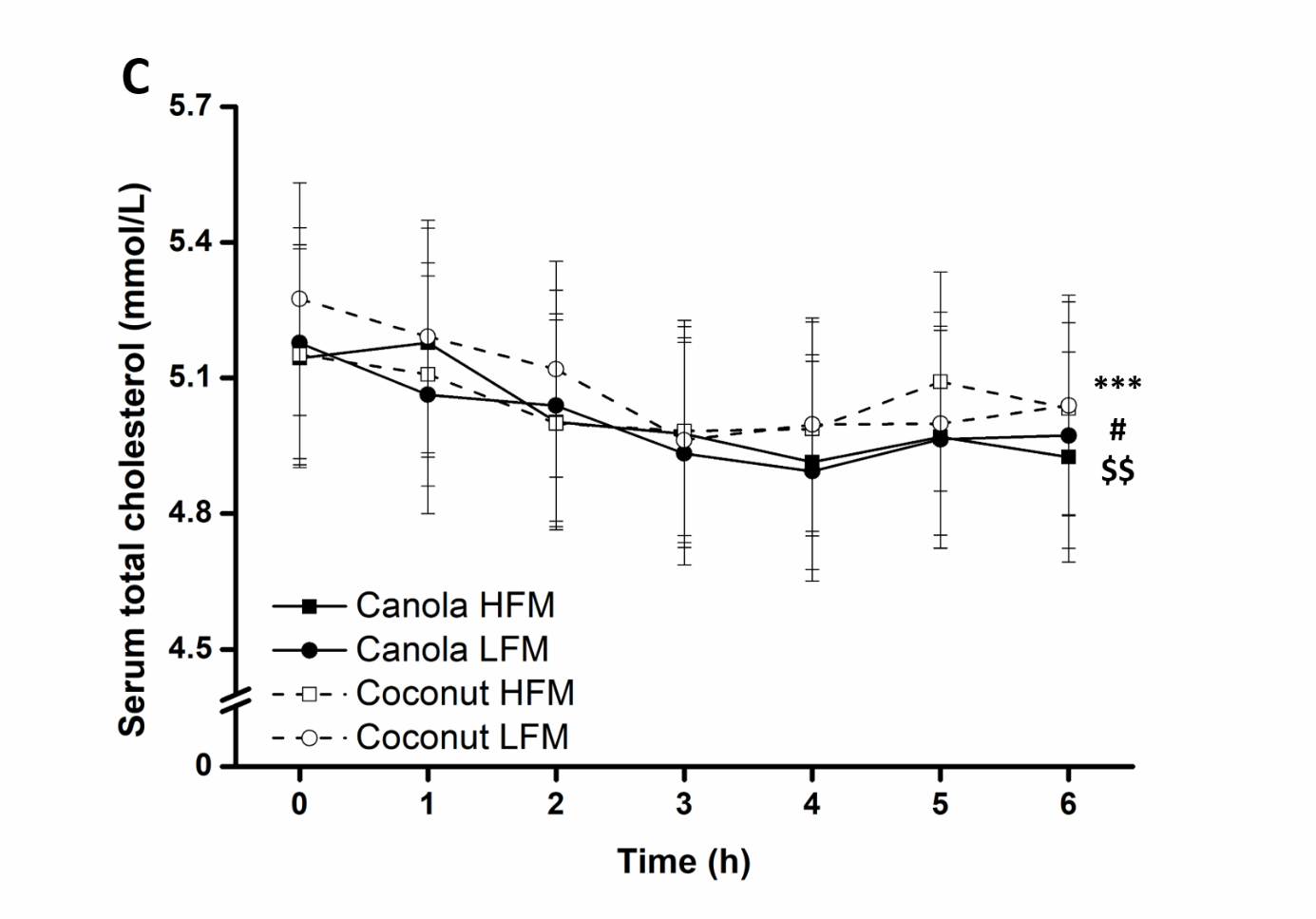


**SUPPLEMENTARY FIGURE 2.** Fasting and postprandial serum concentrations of serum HDL cholesterol (A), serum LDL cholesterol (B), and serum total cholesterol in response to test meals. Data are shown as mean ± SEM (*n* = 29). A linear mixed model with repeated measures was used to test for effects of interventions, time points, and their interactions. ****P* < 0.001 for fixed factor time, #*P* < 0.05 for fixed factor fat amount, §*P* < 0.05 for fat type x time interaction, $$*P* < 0.01 for fat amount x time interaction. Abbreviations: HFM, high-fat meal; LFM, low-fat meal.

**
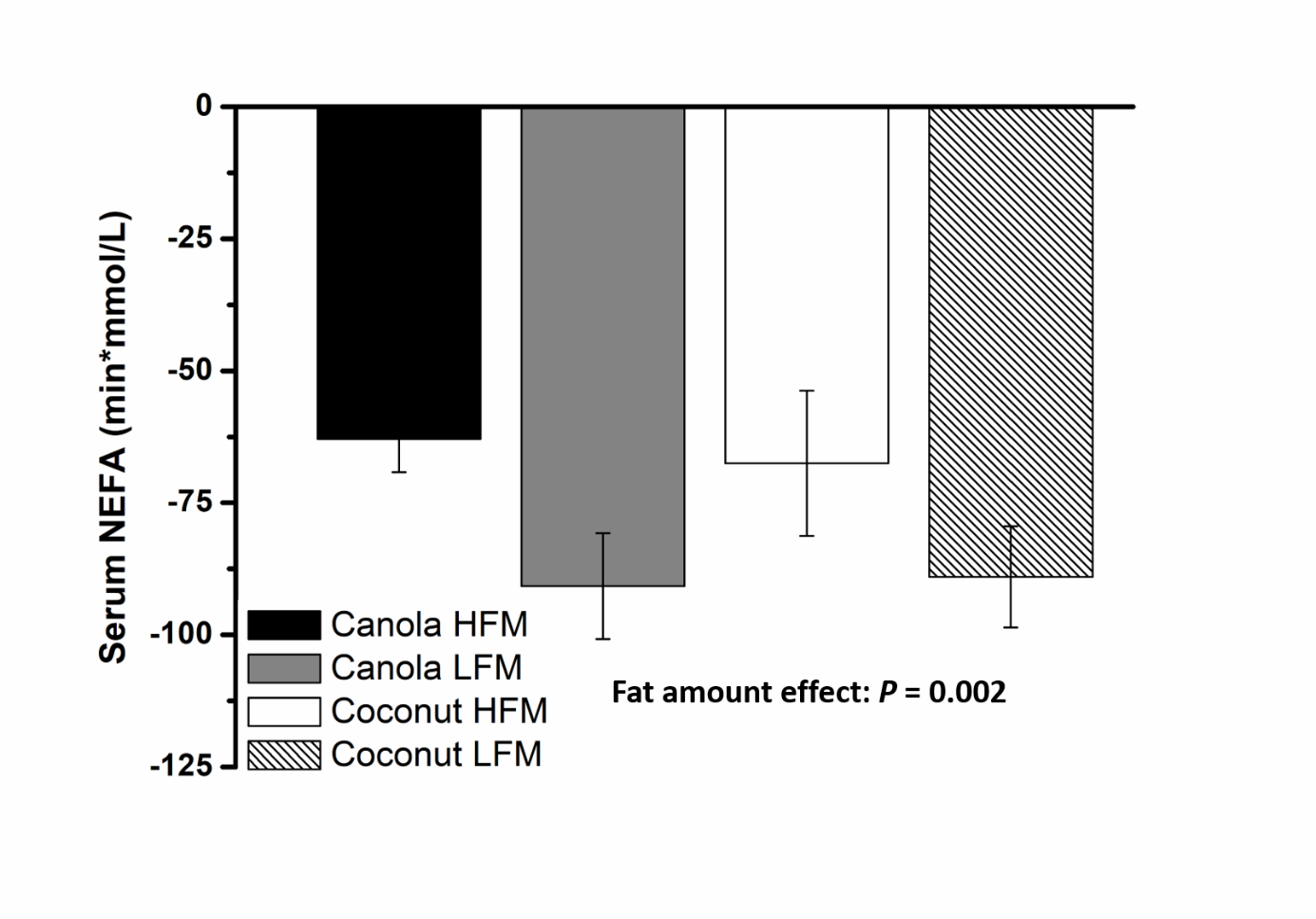
**

**SUPPLEMENTARY FIGURE 3.** Effects of test meals on postprandial concentrations of serum non-esterified fatty acids shown by incremental area under the curve. Data are shown as mean ± SEM (*n* = 29). A linear mixed model was used to test for effects of interventions, time points, and their interactions. Abbreviations: HFM, high-fat meal; LFM, low-fat meal; NEFA, non-esterified fatty acid.

**
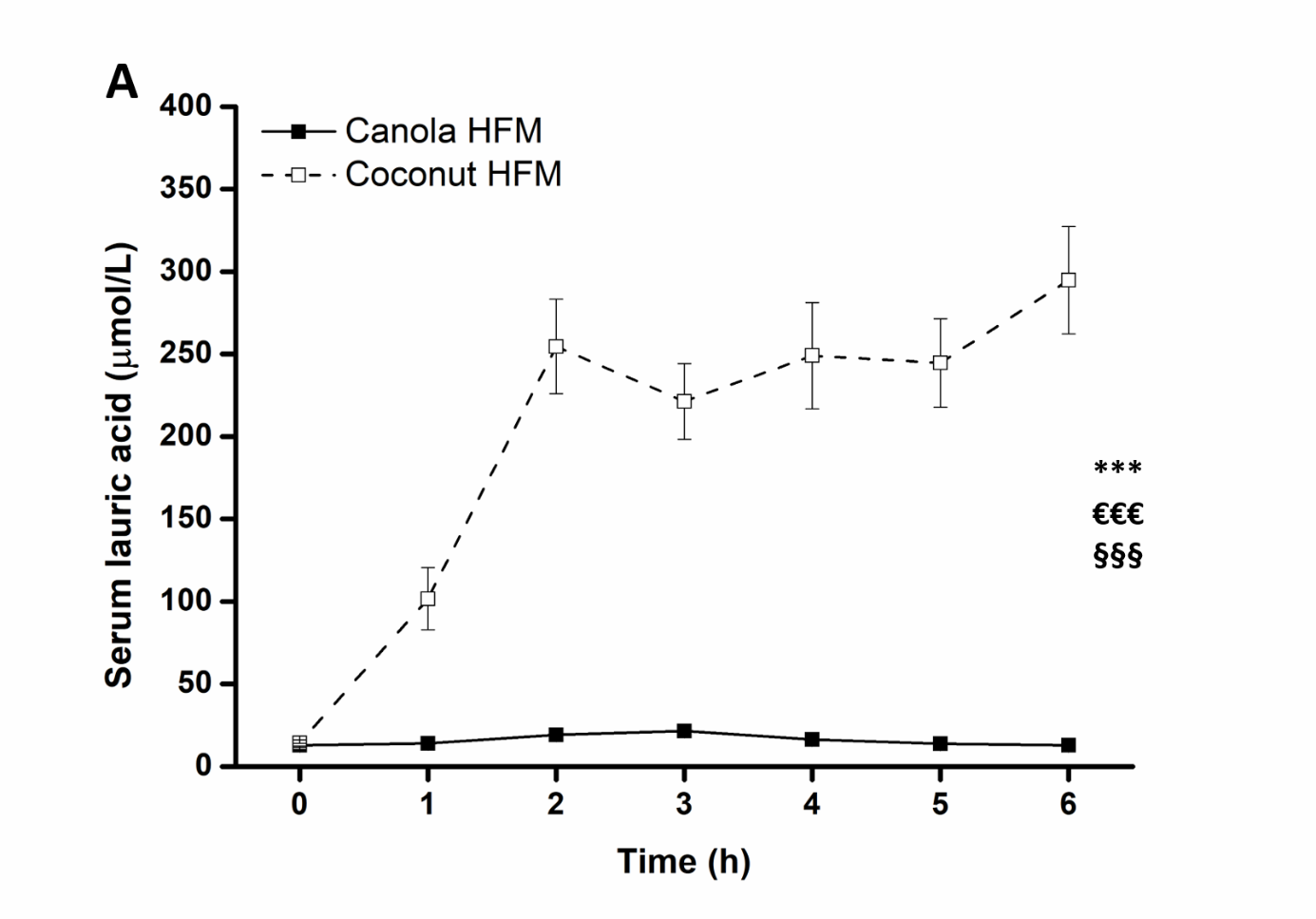
**

**
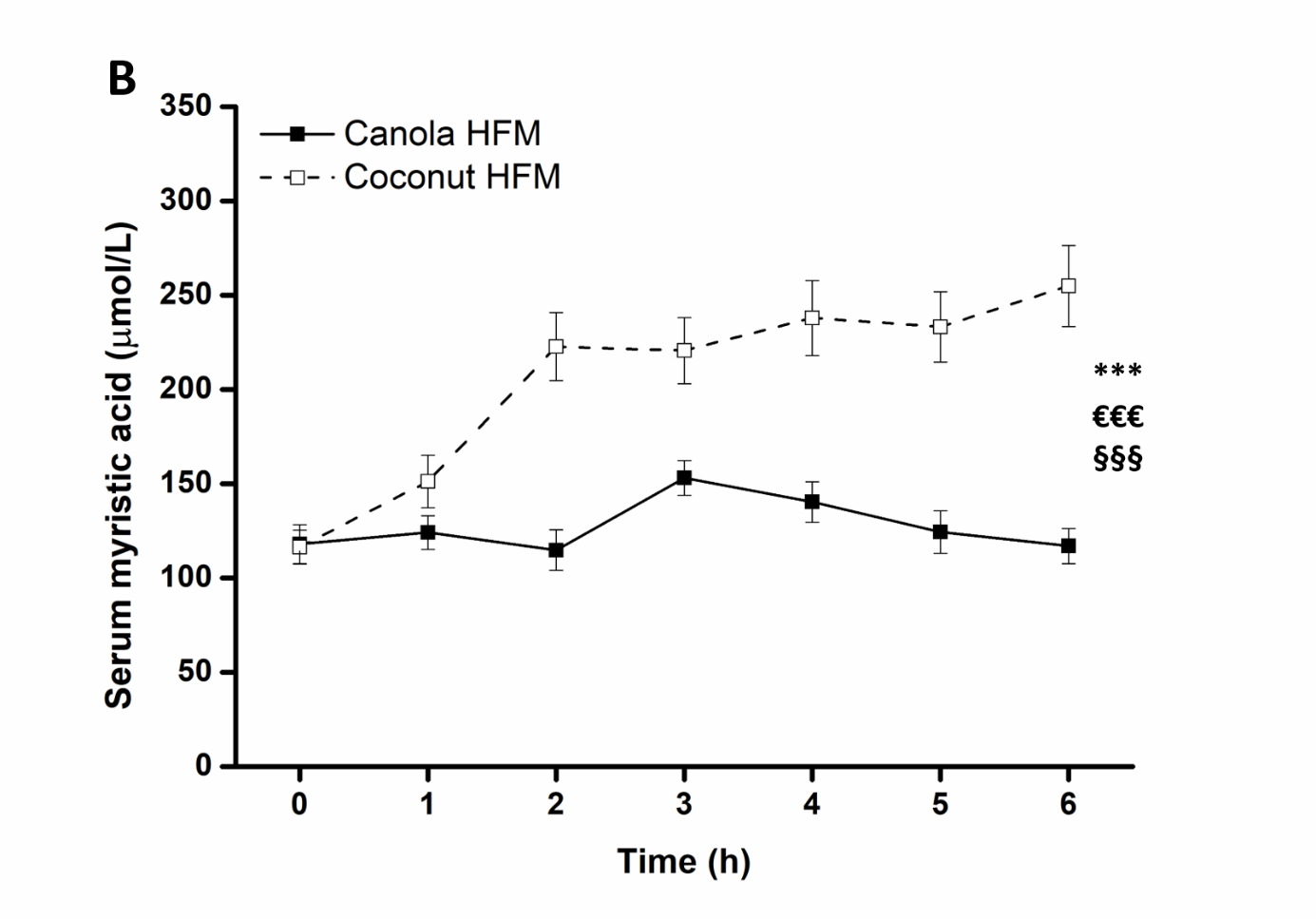
**

**
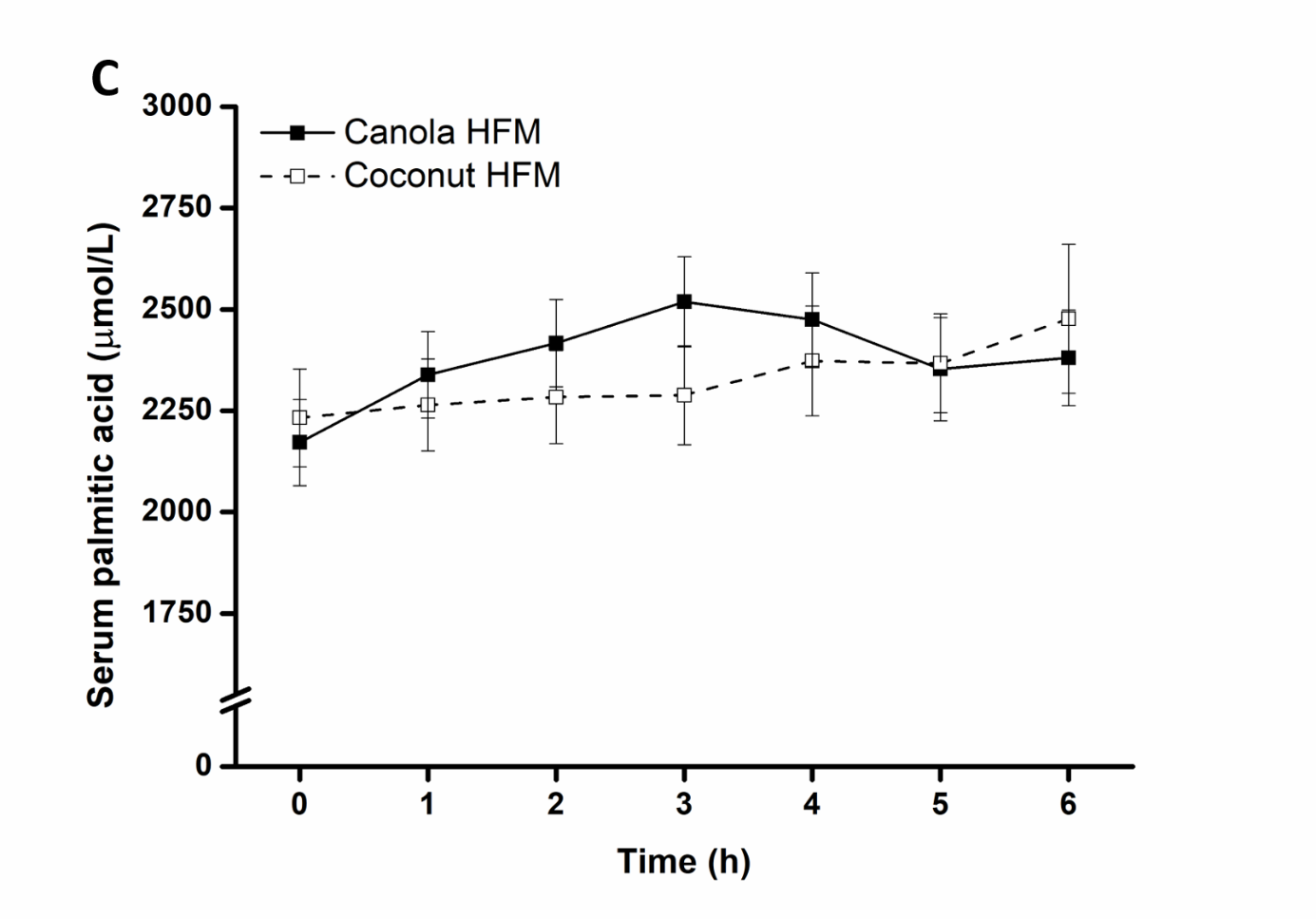
**

**
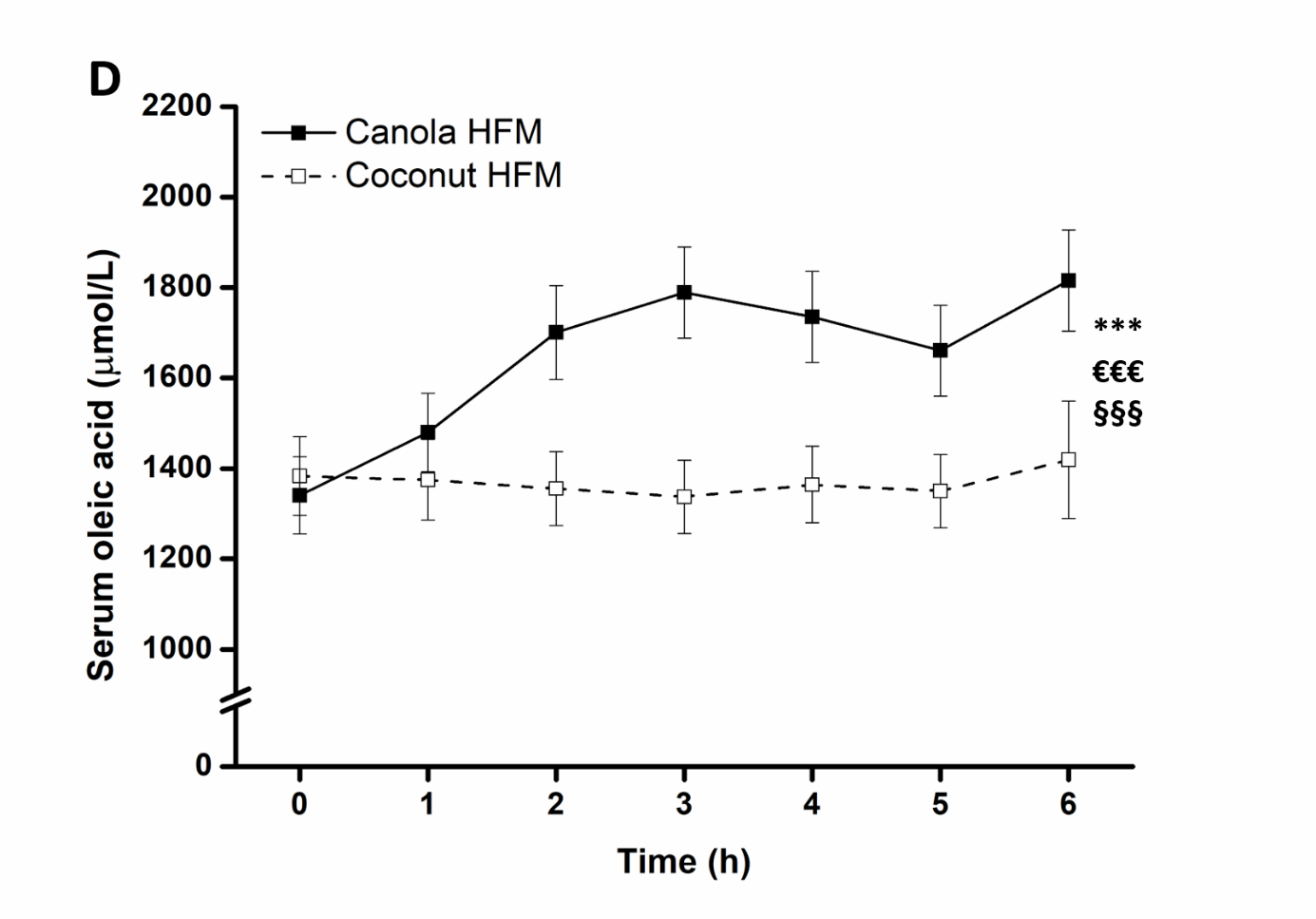
**

**
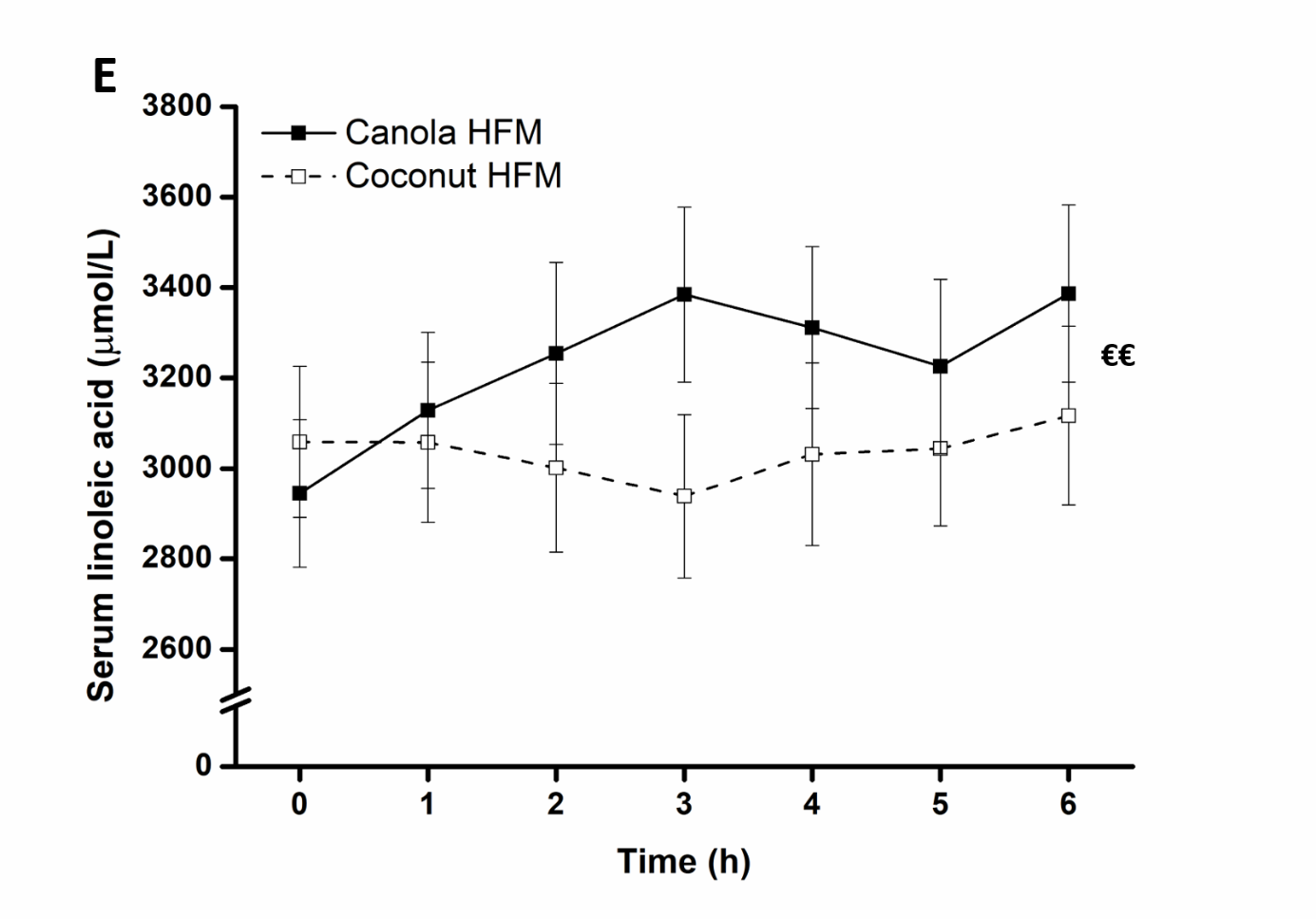
**

**
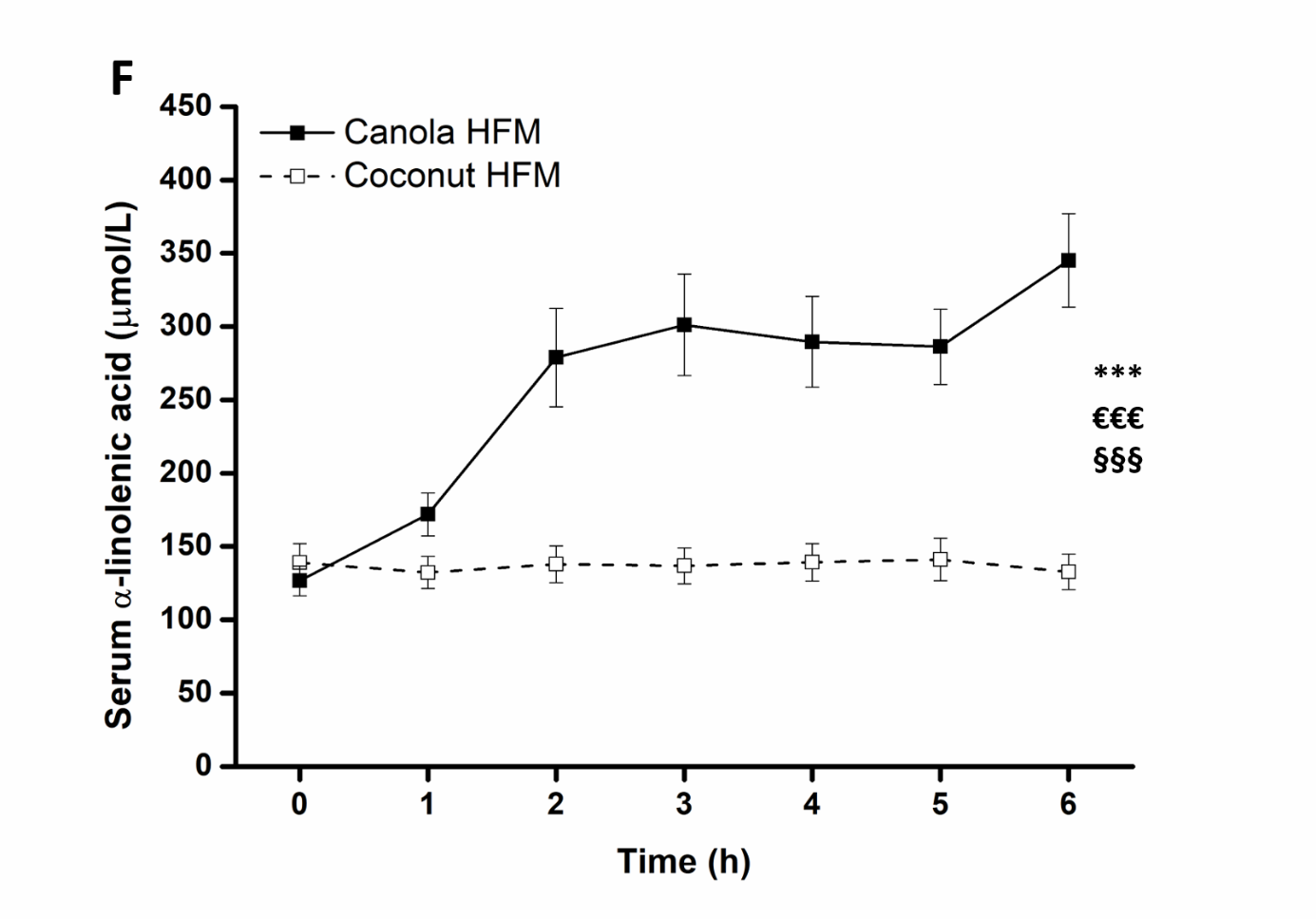
**

**SUPPLEMENTARY FIGURE 4.** Fasting and postprandial serum concentrations of lauric acid (A), myristic acid (B), palmitic acid (C), oleic acid (D), linoleic acid (E), and α-linolenic acid (F) in response to test meals. Data are shown as mean ± SEM (*n* = 29). A linear mixed model with repeated measures was used to test for effects of interventions, time points, and their interactions. ****P* < 0.001 for fixed factor time, €€*P* < 0.01 for fixed factor fat type, €€€*P* < 0.001 for fixed factor fat type, §§§*P* < 0.001 for fat type x time interaction. Abbreviation: HFM, high-fat meal.


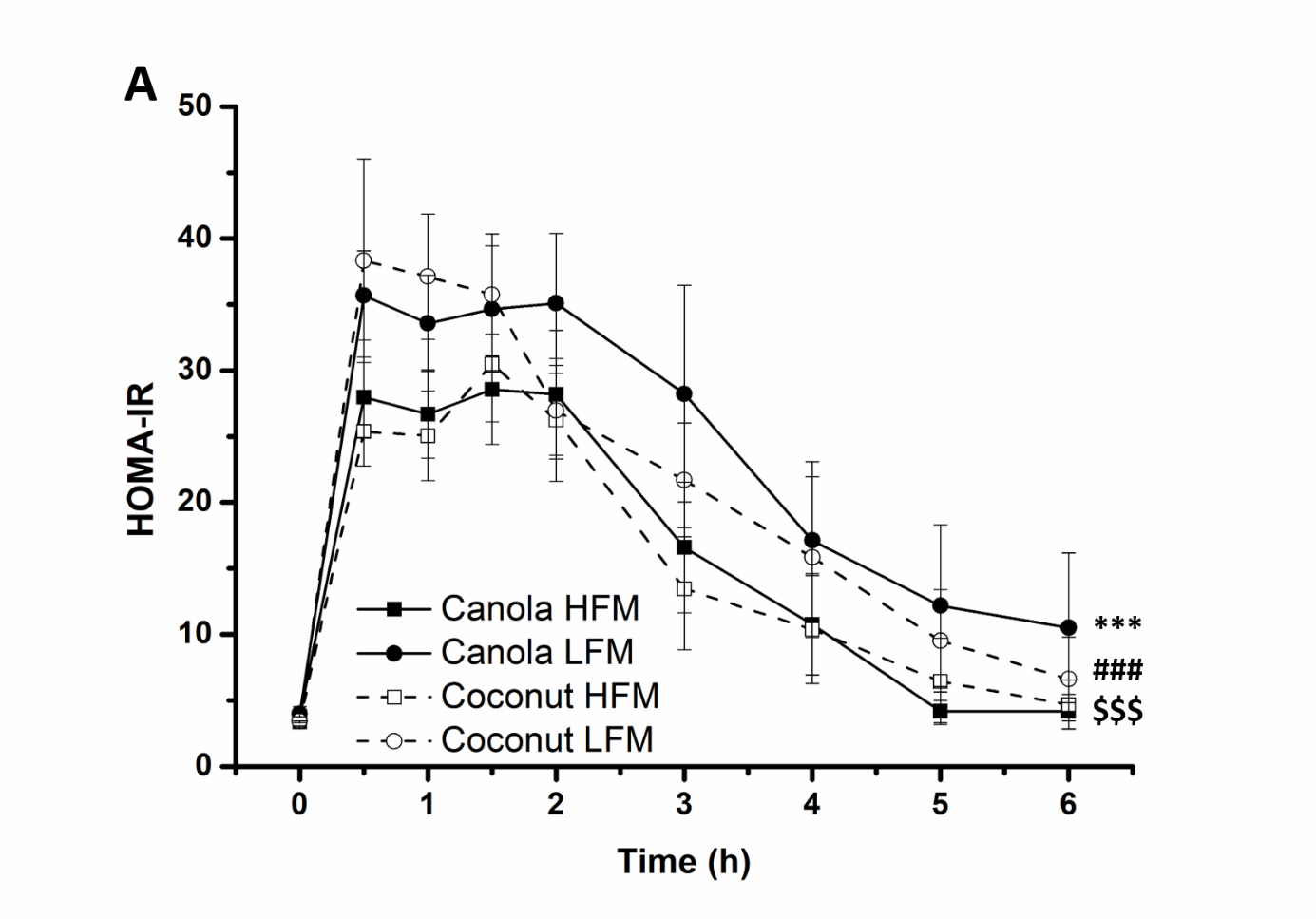


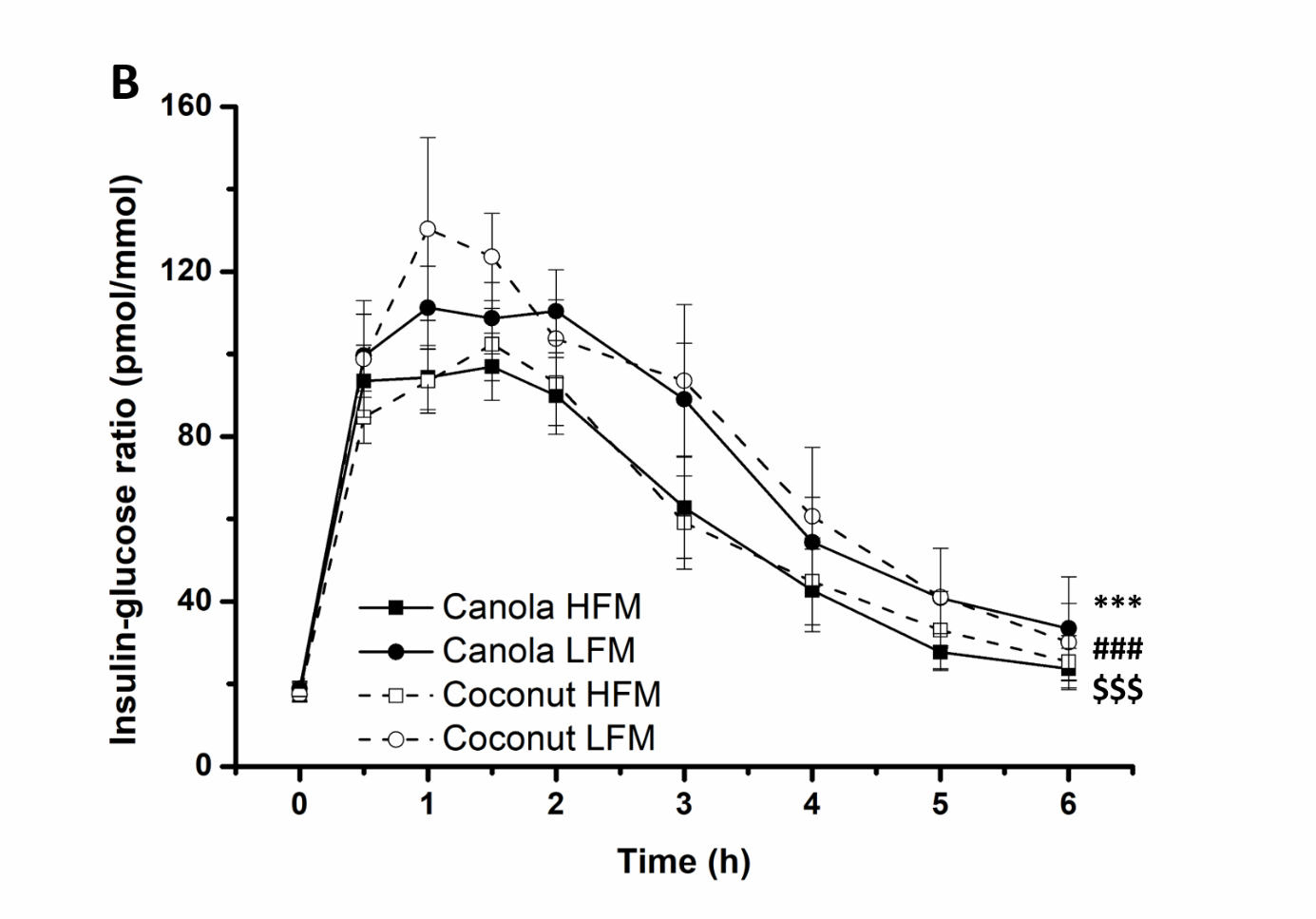


**SUPPLEMENTARY FIGURE 5.** Fasting and postprandial values of homeostasis model assessment for insulin resistance (A) and insulin-glucose ratio (B) in response to test meals. Data are shown as mean ± SEM (*n* = 29). A linear mixed model with repeated measures was used to test for effects of interventions, time points, and their interactions. ****P* < 0.001 for fixed factor time, ###*P* < 0.001 for fixed factor fat amount, $$$*P* < 0.001 for fat amount x time interaction. Abbreviations: HFM, high-fat meal; HOMA-IR, homeostasis model assessment for insulin resistance; LFM, low-fat meal.

**
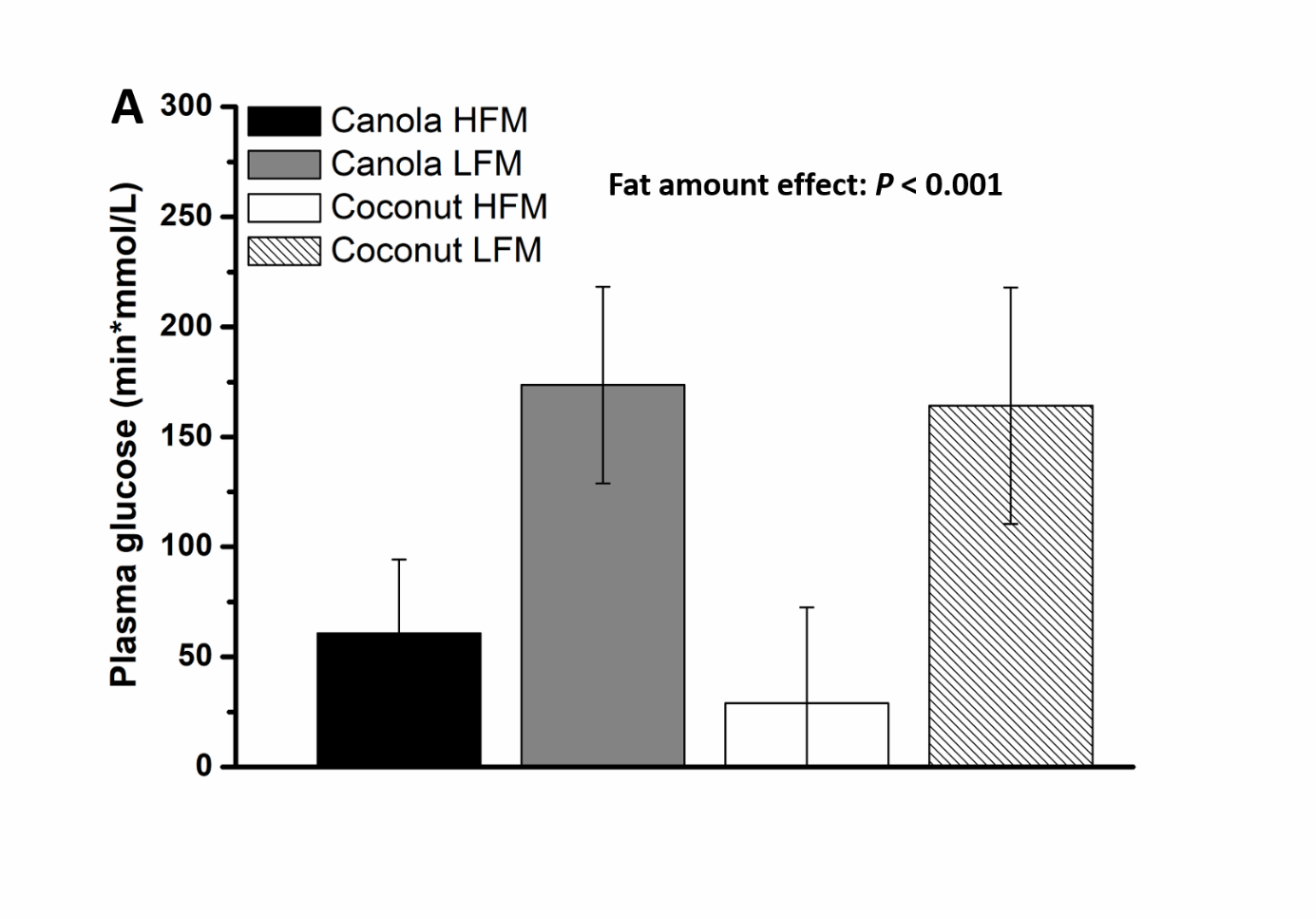
**

**
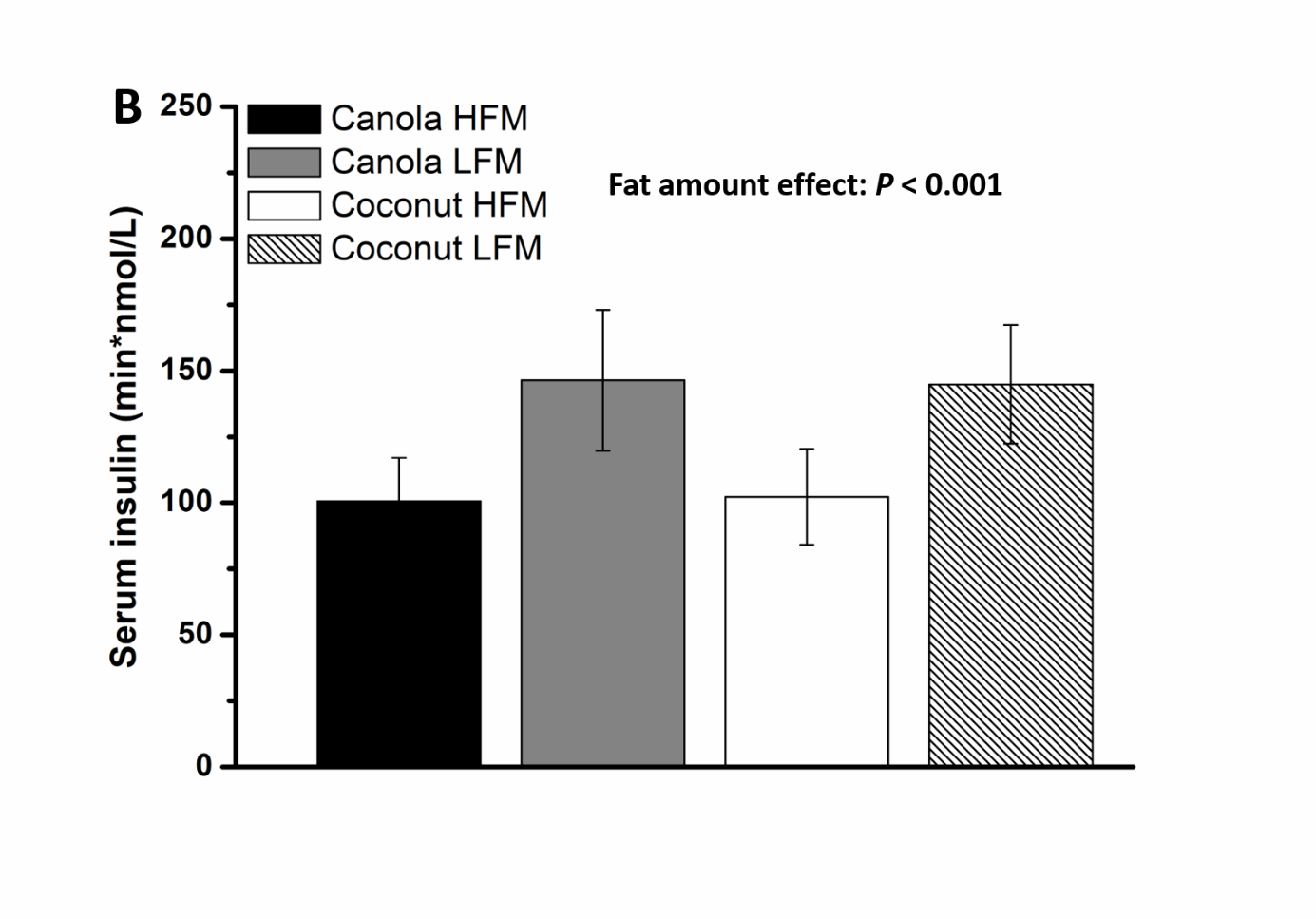
**

**SUPPLEMENTARY FIGURE 6.** Effects of test meals on postprandial concentrations of plasma glucose (A) and serum insulin (B) shown by incremental area under the curve. Data are shown as mean ± SEM (*n* = 29). A linear mixed model was used to test for effects of interventions, time points, and their interactions. Abbreviations: HFM, high-fat meal; LFM, low-fat meal.

**
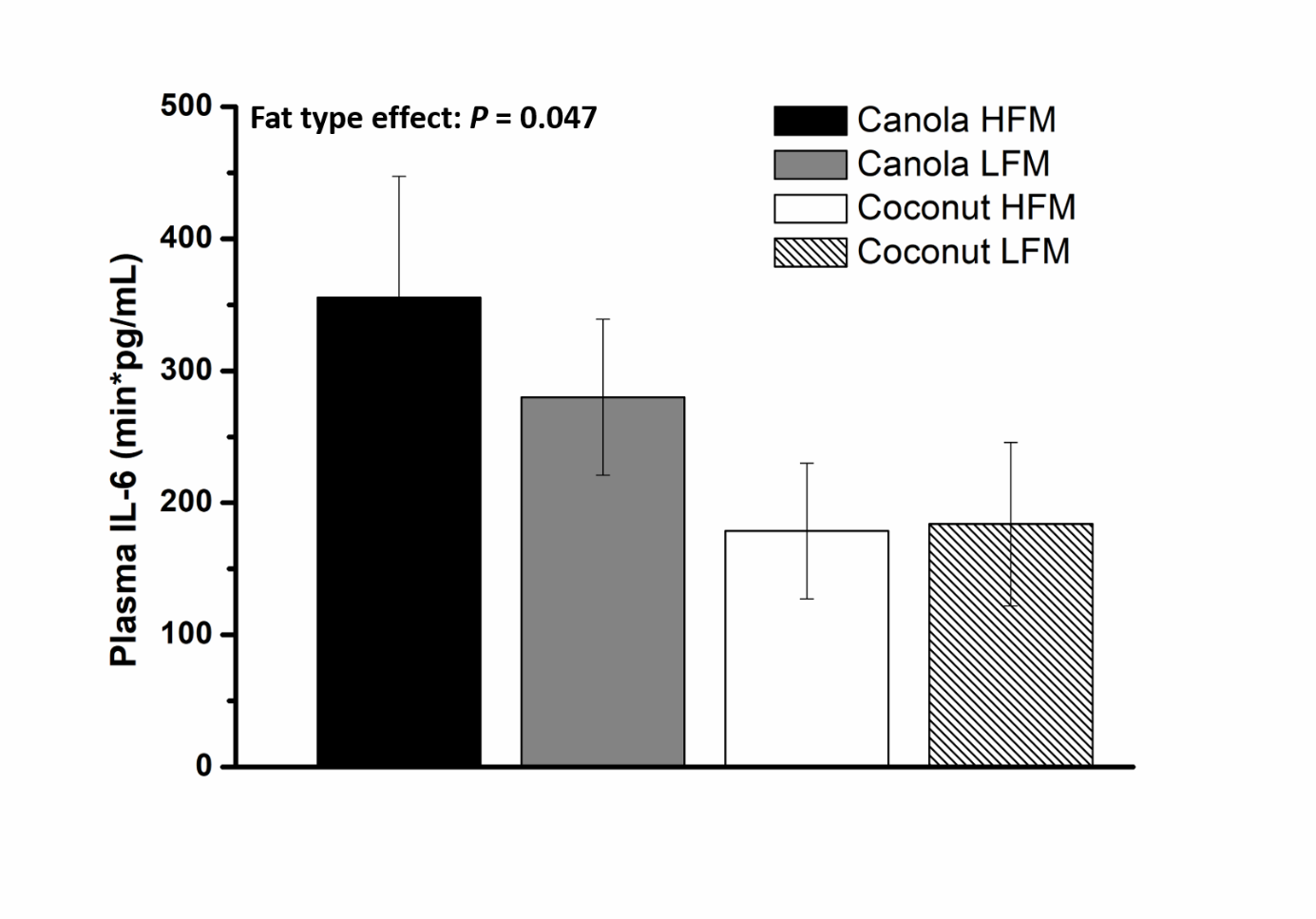
**

**SUPPLEMENTARY FIGURE 7.** Effects of test meals on postprandial concentrations of plasma IL-6 shown by incremental area under the curve. Data are shown as mean ± SEM (*n* = 29). A linear mixed model was used to test for effects of interventions, time points, and their interactions. Abbreviations: HFM, high-fat meal; LFM, low-fat meal.

**
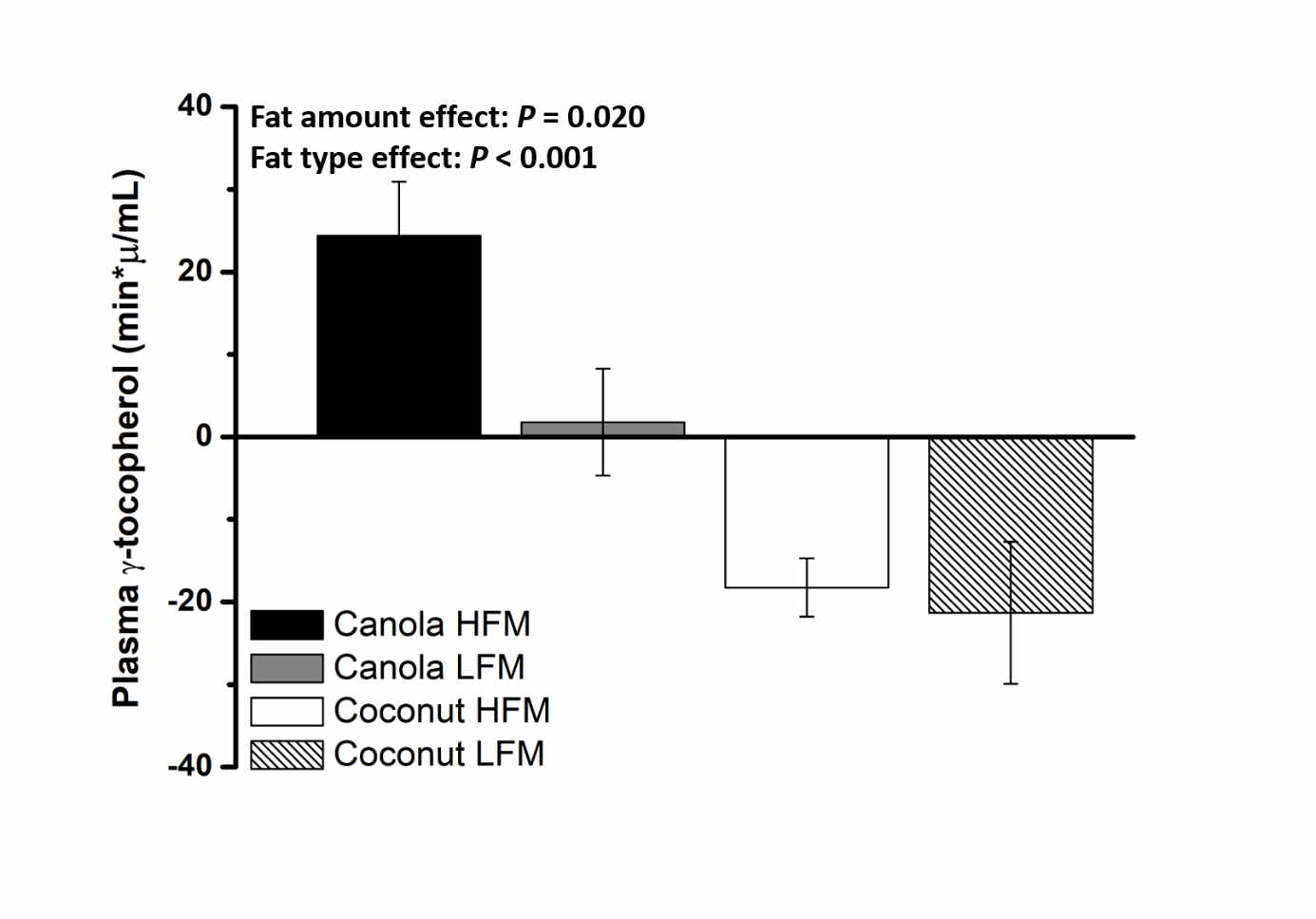
**

**SUPPLEMENTARY FIGURE 8.** Effects of test meals on postprandial concentrations of plasma γ-tocopherol shown by incremental area under the curve. Data are shown as mean ± SEM (*n* = 29). A linear mixed model was used to test for effects of interventions, time points, and their interactions. Abbreviations: HFM, high-fat meal; LFM, low-fat meal.


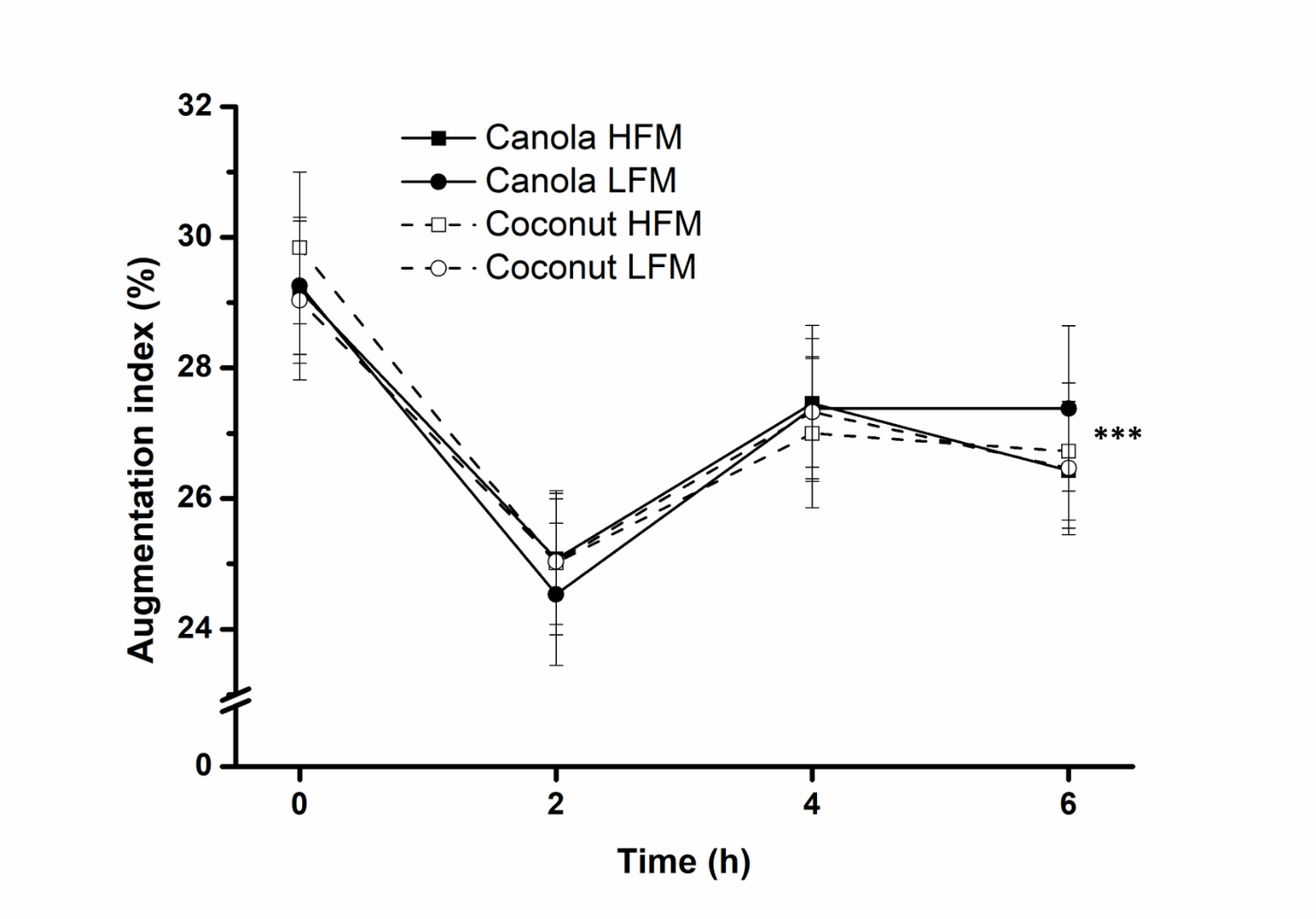


**SUPPLEMENTARY FIGURE 9.** Fasting and postprandial values of augmentation index in response to test meals. Data are shown as mean ± SEM (*n* = 29). A linear mixed model with repeated measures was used to test for effects of interventions, time points, and their interactions. ****P* < 0.001 for fixed factor time. Abbreviations: HFM, high-fat meal; LFM, low-fat meal.
